# Extreme rainfall deficits were not the cause of recurring colonial era famines of southern Indian semi-arid regions

**DOI:** 10.1038/s41598-021-96826-2

**Published:** 2021-09-02

**Authors:** Ranjini Ray, Atreyee Bhattacharya, Gaurav Arora, Kushank Bajaj, Keyle Horton, Shi Chen, Supriyo Chakraborty, Amir Bazaz

**Affiliations:** 1 Indian Institute for Human Settlements (IIHS)-Bengaluru, Karnataka, 560080 India; 2 University of Colorado-Boulder, Boulder, CO 80309 USA; 3 Indraprastha Institute of Information Technology (IIIT)-Delhi, New Delhi, 110020 India; 4 Indian Institute of Tropical Meteorology (IITM), Pune, Maharashtra 411008 India; 5 University of British Columbia, Vancouver, BC V6T1Z4 USA; 6 University of California-Berkeley, Berkeley, CA 94720 USA

**Keywords:** Climate sciences, Palaeoclimate

## Abstract

Using information contained in the eighteenth to twentieth century British administrative documents, preserved in the National Archives of India (NAI), we present a 218-year (1729–1947 AD) record of socioeconomic disruptions and human impacts (famines) associated with ‘rain failures’ that affected the semi-arid regions (SARs) of southern India. By mapping the southern Indian famine record onto long-term spatiotemporal measures of regional rainfall variability, we demonstrate that the SARs of southern India repeatedly experienced famines when annual rainfall reduced by ~ one standard deviation (1 SD), or more, from long-term averages. In other words, ‘rain failures’ listed in the colonial documents as causes of extreme socioeconomic disruptions, food shortages and human distress (famines) in the southern Indian SARs were fluctuations in precipitation well within the normal range of regional rainfall variability and not extreme rainfall deficits (≥ 3 SD). Our study demonstrates that extreme climate events were not necessary conditions for extreme socioeconomic disruptions and human impacts rendered by the colonial era famines in peninsular India. Based on our findings, we suggest that climate change risk assessement should consider the potential impacts of more frequent low-level anomalies (e.g. 1 SD) in drought prone semi-arid regions.

## Introduction

Globally, regions experiencing pronounced impacts of the twenty-first century anthropogenic warming are also some of those experiencing economic stress and social upheavals^1–3^; particularly vulnerable are agricultural societies in semi-arid regions (SARs) of emerging economies^3–5^. Over the past few decades, SARs of peninsular India, which are marked by significant variability in temperature and precipitation^6,7^, have been experiencing frequent meteorological droughts^6,7^; the magnitudes and spatial extent of these droughts have increased due to accelerating climate change^6,7^ that have contributed towards growing socioeconomic disruptions and human impacts in the region^7–12^. To assess the impact of climate variability on human societies, we benchmark the socioeconomic and human history of societies—especially those of SARs societies that are at the frontline of climate risks^6–12^—against independent records of climate variability. There is a vast body of literature, the History of Climate and Society (e.g.^13–17^), which demonstrates that a long-term analysis of climate and human history provides the much-needed context required for a comprehensive assessment of socieoeceonmic disruptions and human impacts arising from climate variability^18,19^. In order to achieve a comprehensive understanding of climate risks in SARs societies^1–21^, especially those in the sub-continent, we assess the role of climate variability in colonial-era famines (extreme food shortages and associated socioeconomic disruptions and human impacts caused by ‘rain failures’) that affected southern India^20,21^.

We extract a 218 year record of socioeconomic disruptions and human impacts (i.e. famines) from eighteenth to twentieth century British administrative records preserved in the National Archives of India (NAI), New Delhi. We benchmark the NAI famine record of India against independent, direct and indirect observations of regional climate (rainfall) variability^22–27^; examples of direct measurements include rain gauge and satellite data^22,23^, whereas indirect observations include paleo proxy based reconstructions of relative humidity, such as, the thickness of tree rings^24,25^ and oxygen isotope (δ^18^O) measurements in speleothems^26,27^. Our goal with the analysis of the NAI famine record in relation to long-term (eighteenth to twentieth century), high-resolution (annual to multidecadal), independent (direct and indirect) regional climate records^22–27^, is to understand the impact of climate variability in the severe socioeconomic outcomes (famines) that were witnessed repeatedly^20,21^ by the southern Indian SARs in the recent past (eighteenth and nineteeth centuries).

## Background

The area of peninsular India bounded between the Balaghat ranges in the north, the Western Ghats (or Shyadris) along the west, and the eastern coast along the east and south is categorized as the semi-arid region or SARs of southern India^6,7,22^ (Figs. S1-S2). Much of the southern Indian SARs (SIS) lies in the rain shadow of the Western Ghats ^6,7,22^ (Figs. S1-S2). The Indian Meteorological Division (IMD) divides the SIS into two climatological zones, namely the ‘western interior’ and the ‘southern interior’^22^ (Figs. S1-S2). The main source of surface water in both these divisions is rainfall, delivered by the Indian monsoon system (ISM), which has two components, namely the incoming southwest ‘summer’ monsoon (June–September) and the retreating southeast ‘winter’ monsoon (October–December)^6,8,22^ (Figs. S1-S2). While the western interior region receives rainfall mainly from the southwest monsoon, the southern interior receives rainfall from both arms of the ISM^6,7,22^ (Figs. S1-S2). The geographical and climatological setting creates a gradient in rainfall from the coast to the interior, with the interiors of both southern and western India receiving between 100 and 600 mm of rainfall annually compared to > 2000 mm of rainfall that the western coastal region receives and ~ 1200 mm of rainfall that the south eastern coast receives^22^ (Figs. S1-S2).

The regional climatology of the SPSs has been well studied; the IMD provides coverage for the SIS (monthly data) since 1870^22^. Furthermore, historical rain gauge data^23^ as well as reconstructions of regional rainfall e.g. tree-ring based reconstructions of relative humidity changes (thicker growth rings indicate higher humidity and by inference higher rainfall^24,25^, (SI section A) and oxygen isotope, δ^18^O, measurements in well-dated (^230^Th), annually resolved speleothems (positive δ^18^O measurements indicate higher relative humidity and by inference higher annual rainfall^26,27^ and SI section A), have shown that the SIS has been semi-arid over the past few centuries with significant variability in sub-decadal and decadal timescales (Figs. S2-S5). Furthermore, the primary mode of rainfall variability in the SIS is 2–3 years, consistent with El-Nino Southern Oscillation (ENSO) phenomenon^28–30^ and 5–7 years consistent with land–atmosphere feedback^31,32^.

As mentioned earlier, there has been significant rainfall variability over the past few decades and recurrance of meteorological droughts^6,7^. Combined with rapid population growth, expansion of water intensive cash crop agriculture (e.g. sugarcane) and urban development^6,7^, these droughts have emerged as major climatological stressors that have led to large-scale socioeconomic disruptions (e.g. crop failures, rising debt, market volatility of cash crops etc.^7–10^) and associated human impacts (e.g. rural–urban migration and farmer suicides^11,12^).

While instrumental measurements^22,23^ and proxy-based reconstructions^24–27^ provide a comprehensive understanding of the regional climate variability over the eighteenth to twentieth centuries, these records by themselves do not provide direct information regarding socioeconomic or human impacts (including disasters) associated with climate variability. Archival documents^16^, which are a type of archive of society^13–17^ contains information that allow us to reconstruct a rich record of socioeconomic changes and human impacts (including but not limited to disasters) related to climate variability^16,17,33–36^. Previous work using human archival documents (both personal diaries and institutional documents) has demonstrated the importance of such material in understanding the human cost associated with climate variability in the context of the subcontinent^16,33–36^ (SI section B).

**Figure 1:**
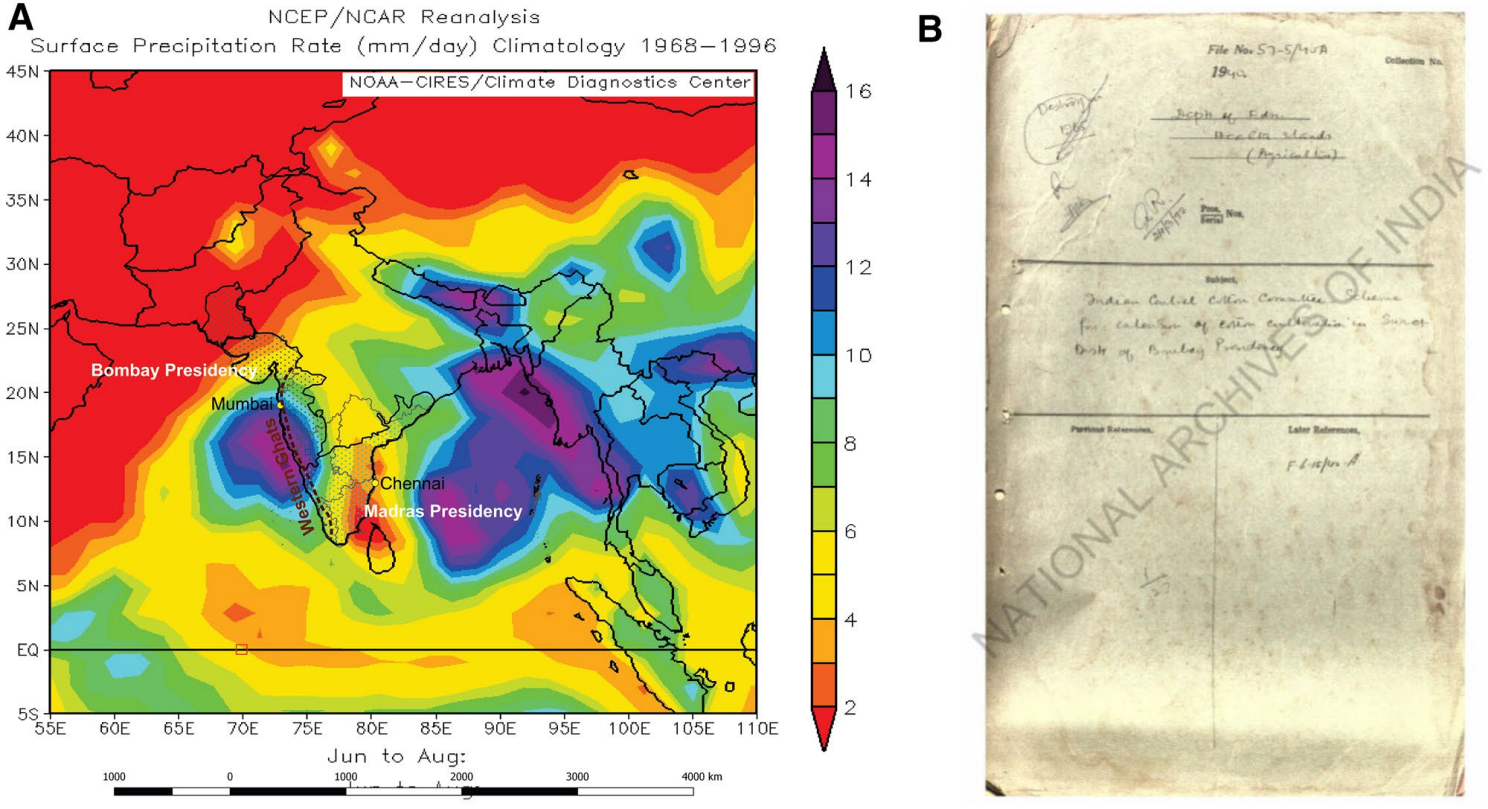
Study area and samples: (**A**) Our study area, the southern semi-arid regions of India (SIS), comprising of the Bombay and Madras Presidencies (administrative subdivisions of British colonial India), overlain on a twentieth century century precipitation climatology map of the Indian subcontinent^42^. Headquartered in the city of Bombay (present day Mumbai) the Bombay presidency included parts of the present day states of Maharashtra, Goa, Gujarat and western Karnataka. Similarly, headquartered in the city of Madras (present day Chennai) the Madras presidency included the present day states of Tamil Nadu, Kerala and parts of Andhra Pradesh, Karnataka and Orissa. There is a rainfall gradient from coast to interiors in both the presidencies (Figs. S1-S2). (**B**) Example of an administrative document preserved in the National Archives of India (NAI), New Delhi; photocopy of a hand written document.

Here we use British administrative documents, pertaining to the period 1729–1947 AD (218 years), preserved in the National Archives of India (NAI) to reconstruct an eighteenth to twentieth century record of socioeconomic and human impacts (famines) associated with ‘rain failures’ events that occurred in the SIS during this period (Fig. 1, SI-Section B). Between the eighteenth to twentieth centuries, the northern hemisphere transitioned from the cooler climate regimes of the Little Ice Age (LIA) to the early decades of the current regime of anthropogenic warming^37,38^. The LIA witnessed frequent incidences of droughts and famines across the northern hemisphere, including in the Indian subcontinent^20,26,27,37–39^, which have been linked to the expansion of the European colonial economy^40,41^. Our large study area (comprising the SS) and long coverage (218 years stretching from the end of the LIA through the early decades of anthropogenic warming), allow us to conduct a robust analysis of the impact of climate variability on the socioeconomic apparatus in colonial SARs societies of southern India.

## Materials and methods

The SIS was governed as two British administrative units, namely, the Madras Presidency and the Bombay Presidency (Fig. 1 and SI section-B). The period between 1729 and 1947 AD can be divided into two parts, namely the Company period (1729–1858 AD), when the two presidencies were governed by the East India Company^43^ (SI Sections A and B) and the Crown period (between 1858 and 1947 AD), when the two presidencies were governed by the British government under the sovereign (^43^ and SI Sections A and B). We have reviewed over 600 colonial administrative documents preserved in the Public Records, Microfilms and Private papers sections of the National Archives of India (NAI), New Delhi. These NAI documents consist of official letters, meeting minutes and correspondences that were used for exchanging information related to financial and administrative management (of Bombay and Madras Presidencies between 1729 and 1947 AD) between district level tax collectors and senior management headquartered in Bombay, Madras and London (Fig. 1 and SI Section B-C). During the Company period, the preserved documents represent mostly handwritten, original copies of letters (Fig. 1 and SI Section B-C), whereas those during the Crown period contain both printed and handwritten documents (Fig. 1 and SI Section B-C).These documents contain a wide range of information (including but not limited to meteorological information, which is discussed in detail in the Results section) related to district or sub-district level administrative information, much of which focuses on details pertaining to revenue collection (or non-collection) from land-based agricultural production.

We found a total of 60 documents, all in the Public Record holdings (Land Revenue, Agriculture, Home and Medical department), which contain information pertaining to climate and impacts in the Madras and Bombay presidencies of the SIS between 1729 and 1840 AD. During the Company period, information—mostly in the form of exchanges between local administrators and senior officials regarding financial matters related to climate disasters, impacts and relief measures in Bombay and Madras Presidencies (Fig. 1 and SI Sections B-C)—were contained in the documents of the Land Revenue and Agriculture section of the Home department. During the first two decades of the Crown Period (1858–1871), most of the documents pertaining to climate and impacts belonged in the Home Department. From 1871, the Department of Revenue and Agriculture and Commerce was set up to handle all matters related to agriculture in the Indian subcontinent. In 1881, the Department of Revenue & Agriculture was set up to manage combined portfolios of education, health, agriculture and revenue (until Independence of India in 1947). Furthermore, the documents in the Medical Department contained information pertaining to epidemics during climate disasters. In addition, printed reports, commissioned by the British Government became common during the Crown period; these reports also contained information regarding climate and impacts in the two presidencies. We extract information pertaining to the disasters associated with climate, listed causes and impacts associated with disasters from these documents and compare the information against independent measures^17–27^ of climate variability to assess the possible role of climate variability in climate disasters.

## Results

**Figure 2:**
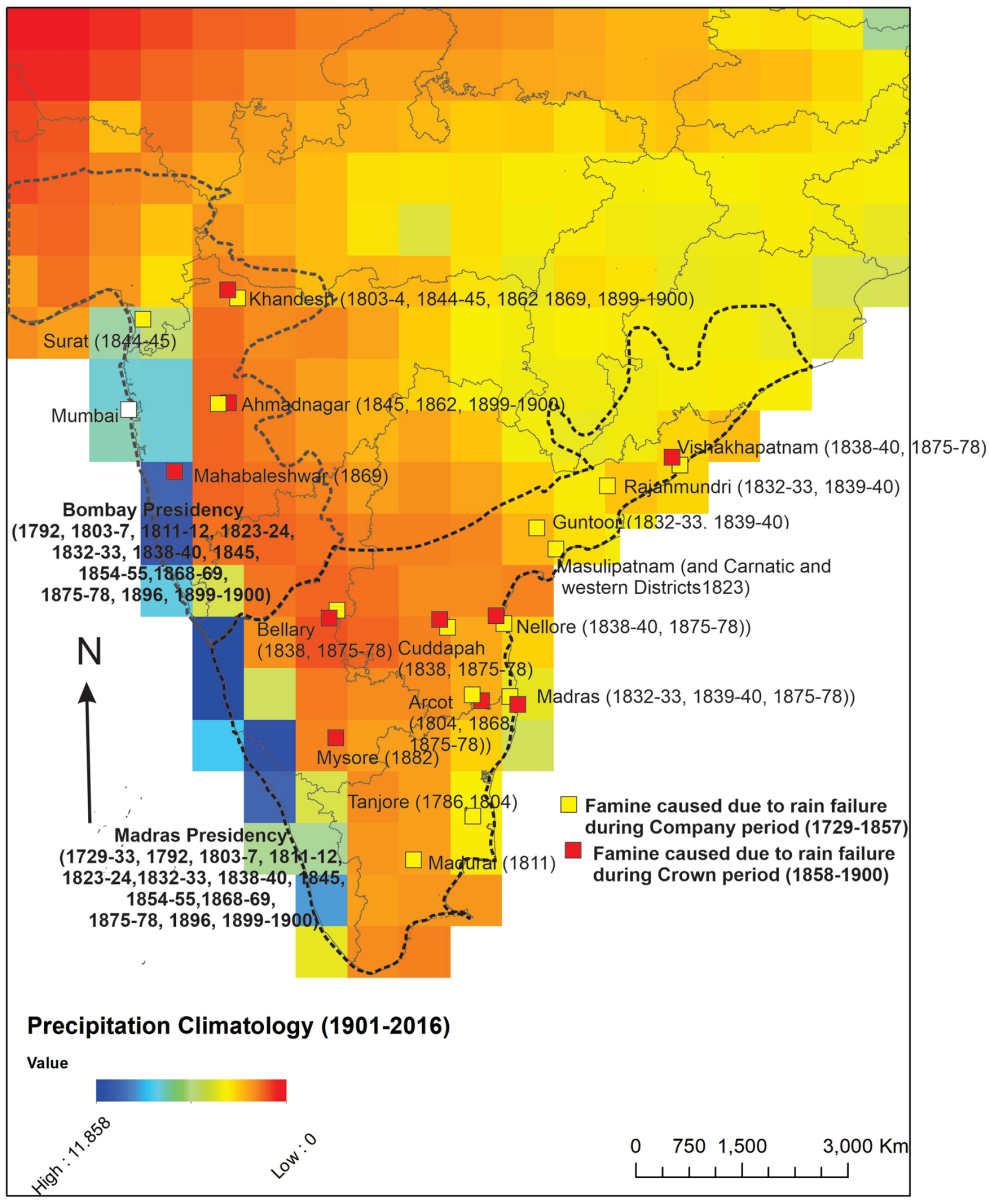
Semi-arid regions of peninsular India at-risk of socioeconomic disruptions associated with rain failures: A map depicting the location of famines that affected southern India during the colonial period (1729–1947 AD); the famine-affected localities are overlain on a 115-year (1901–2015) precipitation climatology map of India (Indian Meteorology Division i.e. the IMD^22^). The colonial-era administrative documents mostly report the incidence of famines in terms of presidencies i.e. in Bombay and/or Madras Presidencies. For example the famines of 1729–1733, 1792, 1803–1807, 1811–1812, 1823–1824, 1832–1833, 1838–1840, 1845, 1854–1855, 1868–1869, 1875–1878, 1896, 1899–1900 were reported in documents pertaining to both Bombay and Madras presidencies (note that the famines of 1792, 1803–1807, 1811–1812, 1823–1824, 1832–1833, 1838–1840, 1845, 1854–1855 of Bombay Presidency were also reported by^34,35^). However, some of the documents we reviewed provide additional details i.e. includes the name of the impacted districts (most likely reflecting either the administrative requirement for such details or the tendency of specific administrative officials to include details). The famines with information regarding affected districts are represented as squares (along with the name of the district); yellow squares denote the locations of famines that occurred during the East India Company period (1729–1857) and the red squares denote the locations of famines during Crown period (1858–1947). During both periods, the same numbers of famines were reported from both presidencies. All the famines reported for the two peninsular Indian presidencies for the British Colonial period (> 200 years) are located in semi-arid regions (Figs. 1–2, S1-S5), establishing that semi-arid regions are most prone to socioeconomic disruptions, embodied as famines, caused by rain deficiencies, embodied as ‘rain failures’ in the colonial era documents. The fact that famines crosscut both presidencies and administrative regimes underscore the fact that semi-arid regions of peninsular India are prone to socioeconomic disruptions associated with rain failures. Note, that there was no mention of famines after 1901, i.e. after the final report by the Famine Commission (1901).

Our results are listed in Table S1 and depicted in Fig. 2. Here we discuss the salient features of the table (S1) under the following categories.

### Climate information contained in the NAI documents

First, the climate attribute that finds mention in the documents of both the Company and the Crown periods is rainfall variability i.e. the amount of rainfall in qualitative terms (less vs. more), disasters that occurred due to rainfall fluctuations (rain failure vs. deluge) and impacts of rainfall fluctuations (famines vs. floods). Second, the documents contain a detailed description and chronological sequences of socioeconomic disruptions and human impacts following rainfall fluctuations. Third, the documents provide information related to occurrence and impacts of rainfall fluctuations at the local (district) and regional (presidency) levels. We extracted information regarding rainfall fluctuations (location and timing) and impacts (in terms of districts or presidencies impacted, the nature of impact, and the chronological sequence of the impacts) from the NAI documents for the period 1729–1947.

### Record of ‘rain failures’

Barring the mention of two local flooding events in the Bombay presidency (Khandesh in 1839 and Kaira of Gujarat in 1840), all mentions of rainfall fluctuations were related to the timing, location, duration and impacts of ‘rain failure’, collectively termed as ‘famines caused by rain failures’, which impacted Bombay and Madras Presidencies between eighteenth to twentieth centuries (Table S1 and Fig. 2). The term ‘famine’ was used in all instances to indicate a condition of acute food shortage and a hike in food grain prices. Since the earliest instances of British administrative presence in southern India was from the second and third decades of the eighteenth century^43^ (SI section B), there are only a handful of administrative documents that pertain to the early part of the eighteenth century and those that exist do not have any mention of the term ‘famine’. We encounter the term ‘famines’ in relation to SIS from the late 1720s e.g. famines in the Madras presidency during 1729–1733 and 1792 (Table S1 and Fig. 2).

In the nineteenth century records pertaining to both Bombay and Madras Presidencies, we encounter the term famines and abundant discussions related to famines caused by rain failure. Especially discussed were famines due to rain failures of 1803–1807, 1811–1812, 1823–1824, 1832–1833, 1838–1840, 1868–1869, 1875–1878, 1896 and 1899–1900 (Table S1 and Fig. 2). These famines affected several districts of both Madras and Bombay presidencies and usually lasted for 3–4 years. Two of these famines deserve special mention i.e. the Great Famine of 1875–1878 and the Indian Famine of 1899–1900. Both were caused by ‘rain failure’ (as mentioned in the NAI documents) in which millions of people perished^20,21,43^. In addition to the above, we found mention of famines of 1845 (in Gujarat), 1854–1855 (parts of Bombay and Madras Presidencies), 1862 (Ahmednagar) and 1882 (Mysore) (Table S1 and Fig. 2). The famine of 1875–1876 prompted the formulation of the Famine Commission in 1880^44^; the last report by the commission is in 1901. Thereafter, we find no mention of famines in the British Administrative documents in the NAI pertaining to twentyteenth century in peninsular India. However, we found newspaper clippings (Amrita Bazar Patrika Calcutta, 7-9-1918) in the NAI that reported a food riot in Madurai (in Madras Presidency) in 1918, but no mention of famines. Overall, in both Crown and Company periods, the same numbers of famines were reported from both presidencies.

### Impacts of rain failures

The eighteenth to twentieth century British Administrative documents (correspondences and reports) archived at the NAI, contains vivid descriptions of socioeconomic disruptions and human impacts during the famines caused by ‘rain failures’ (Table S1). Particularly common during the famines were crop failures, price hikes, starvation and farmer migration (Table S1). Furthermore, these documents contain a chronological sequence of socioeconomic stress triggered by ‘rain failures’; the sequence started with ‘rain failure’ that last for at least one, but often two consecutive year(s) leading to crop failures followed by the grain price hike, food scarcity and farmer migration (both temporary and permanent) in that order. In addition, we find documentation of an increase in starvation and mortality, especially during periods when there was no alternate employment (usually in road building along urban corridors). We also find mention of increased migration, the incidence of slavery (including selling children), epidemics, riots and death in the NAI documents (Table S1). The socioeconomic disruptions and human impacts (during acute food shortages and rise of food grain prices together characterized as ‘famines’) occurred in both presidencies during both the Company and Crown Period up to 1899 (Table S1).

## Discussions

Based on the duration and spatial scale over which socioeconomic disruption and human impacts (famines) persisted, we classify the NAI famines into two categories, namely ‘regional’ and ‘local’. We define “regional famines” to be those that affected more than one district (usually several districts in both the Bombay and Madras Presidencies), whereas we define “local famines” as those whose impact was restricted to one district. The classification scheme allows us to comprehensively assess the climatological context of these SIS famines.

**Figure 3:**
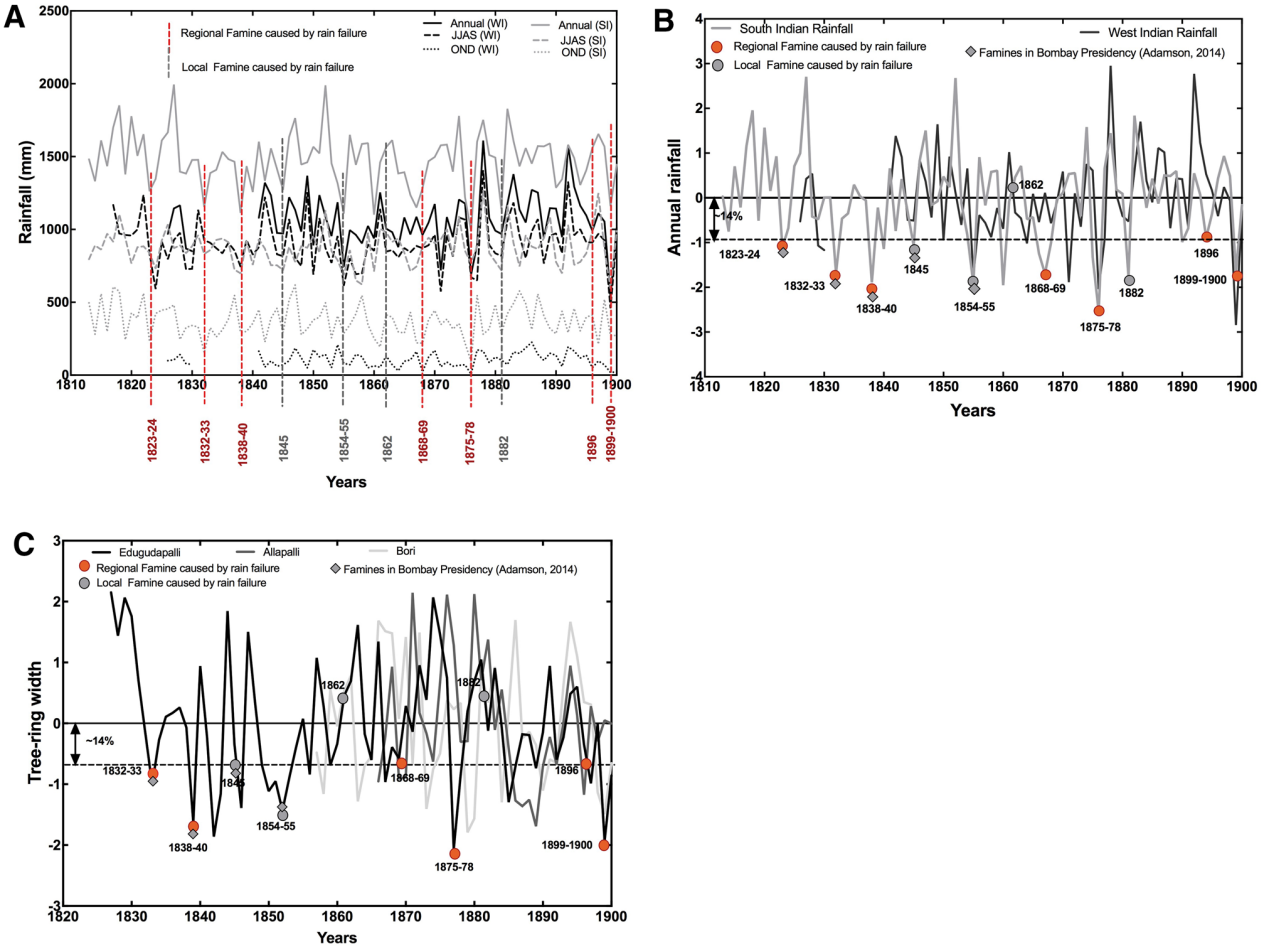
The relationship between rainfall variability and famines: (**A**) Historical famines (including those reported before^33–36^) plotted against historical instrumental rainfall data^23^, where the authors collated historical instrumental data for the period 1813–2005 for seven zones in India (Fig. S3), including the southern and western India, using 316 well-spread station data for monsoon i.e. June–July–August–September (JJAS), post monsoon i.e. October–November–December (OND) and annual averages. (**B**) Relationship between the occurrence of famines and historical instrumental rainfall data^23^ converted to deviations from the series average (also refer to Fig. S3). (**C**) Relationship between the occurrence of famines and tree-ring based century-scale reconstructions, converted to deviations from the series average. The three locations of the tree ring sites i.e. Edugudapalli and Allapalli in Telengana and Bori in Madhya Pradesh, are all in the interiors of peninsular India and representative of regional rainfall variations^24,25^ (also see Fig. S3). In most instances, when rainfall dropped below 1 standard deviation (SD), we find that famines occur (usually regional, denoted by red circles) in the semi-arid regions of peninsular India. The reported famines occur when both JJAS and OND monsoons fell below normal by more than 1 SD (as evident in the analysis of famines in relation to the historical instrument as well as tree-ring based reconstructions) and such deviations tend to occur at (or close to) the minima (dry) of the rainfall variability experienced by peninsular India. The famine of 1862 was an exception in the sense that it was a local one in the northwest part of the Bombay presidency when June–July–August September i.e. JJAS rainfall was lower than average in western India but rainfall was normal in southern India. We suggest that the 1 SD deviation represents a threshold that is breached between 5–10 years due to climate variability occurring as an interplay of ISM, IOD and land–atmosphere feedback^28–32,45^.

Our results indicate that parts of the Indian peninsular SARs suffered severe socioeconomic disruptions and human impacts (famines), both locally and regionally, from ‘rain failures’ (Table S1 and Fig. 2). In addition, eastern coastal areas of peninsular India (in the Madras Presidency), which suffered famines during 1832–1833, 1838–1839, 1875–1878, 1899–1900 (Table S1 and Fig. 2), were also susceptible to severe impacts from ‘rain failures’, at both local and regional scales. Second, colonial administrators point to ‘rain failures’ as causes of severe socioeconomic disruptions and human impacts (famines) that affected peninsular Indian SARs over the eighteenth to twentieth centuries; we find that regional famines occurred when ‘rain failure’ persisted for two years (reduction summer rainfall in the Bombay Presidency and reduction of both summer and winter rainfall in the Madras Presidency, Fig. 3A) and local famines occurred when ‘rain failure’ persisted over the course of a year or lesser (usually reduction of the summer rainfall in both presidencies, Fig. 3A). It is important to note that local (district level) manifestations of reduced summer rainfall may vary significantly and local famines could be associated with more severe rain deficits.

To assess the critical term ‘rain failures’, which colonial administrators point to as the cause behind socioeconomic disruptions and human impacts at local and regional scales, we compared the famine record of Indian peninsular SARs (which included the famine record that we retrieved from the NAI British administrative documents as well as previously published records of famines in the two presidencies^33–36^ and SI-Section-B), to quantitative rainfall data from IMD^22^, historical rain gauges^23^ and proxy based reconstructions of relative humidity (tree ring thickness^24,25^ and δ^18^O in cave carbonates^26,27^).

We find that all regional (and most local) famines occurred when annual rainfall totals deviated from long-term annual averages by ≥ 1 standard deviation (SD) from long-term regional annual average rainfall (Fig. 3B-C, S3-S4). Specifically, most of the famines were associated with a deviation of 1 SD for the southern Interior is ~ 13% and for the western interior is ~ 17% i.e. an average of 14% deviation for the SPS (based on both IMD and historical rain gauge reconstructions^22,23^).

In other words, the term ‘rain-failure’ that colonial administrators during the Company and Crown period (as represented in the eighteenth to twentieth century British administrative documents) listed as the cause of socioeconomic disruptions and human impacts (both regionally and locally) is within the range of rainfall variability inherent to the southern Indian  SARs and not due to climatological extremes i.e. not because of extreme rainfall reductions (> 3 SD from long-term averages^22–26^). In fact, the rainfall reduction threshold for famines and associated impacts appear to be ~ 1 SD of the long-term average^20,21^. In addition, famines occurred repeatedly in both presidencies during both administrative regimes (Company and Crown periods, respectively); this observation underscores that policies and practices, adopted by administrators over two centuries, did not mitigate against the occurrence of famines of semi-arid regions of peninsular India at least till the beginning of the twentieth century.

**Figure 4:**
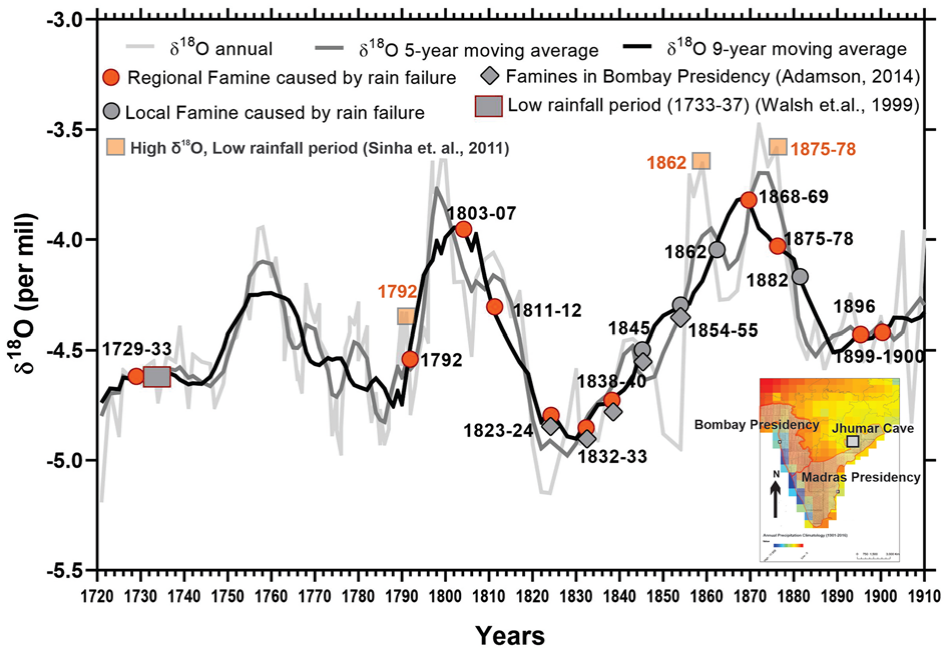
Relationship between long-term rainfall climatology and famines: Analysis of famine incidence and δ^18^O measurements in cave carbonates (also called speleothems); inset shows the location of the Jhumar Cave, Chhattisgarh, the only available long-term and high-resolution record of regional climatology^26,27^. Also see Fig. S5 and SI for details. Higher δ^18^O values indicate a drier period (also see SI section A) and lower values indicate wetter periods. Annual, 5-year and 9-year moving averages are plotted. There is evidence of quasi-multidecadal cyclicity (Fig. S5) in the δ^18^O data, indicating complex climatological controls (both from oceanic as well as land–atmosphere processes and feedbacks) on rainfall variability in the SARs of southern India in addition to the robust ENSO signal^28–30,45^. The cycles, however, are associated with famines, with a majority of the regional famines (in red) occur during the dry cycle (in some cases we note that the famines occur along the ascending limb of the annual data; this seeming offset is because of the memory effect of carbonate depositional process that lag rainfall^26,27,46^). Also, each of the reported famines is associated with higher δ^18^O values (indicating rain deficient year). There is a lack of famines related information in 1720–1790 (documents present are fewer).

We map the Indian peninsular SARs famine record on to δ^18^O measurements in cave carbonates (speleothems) of Jhumar (Fig. 4, S5, SI-Section-A^26,27^), which represent moisture variability (tied to the intensity of the ISM) in the region over the last millenniuum^26,27^. The mapping exercise is intended to allow us to assess the relationship between famine occurrences with regional climatology; the tree rings and instrumental data provide information on concurrent meteorology^22–25^, whereas δ^18^O measurements in speleothems provide a decadal average^26,27,46^. We find that most of the famine occurrences coincide with both the dry and the wet cycles (as evident from the cave δ^18^O record, Fig. 4, SI section-C), although episodes of low rainfall were more frequent during the drier (low moisture) phases of the late nineteenth century. The drier cycles of the late nineteenth century coincided with the transition of the northern hemisphere from the cooler LIA to the warmer decades following the end of the LIA^37,38^ and concurrent expansion and intensification of the British Colonial economy^20,40,41^.

The last decades of the nineteenth century that marked the climate transition from the LIA to the current warming period has witnessed rain failures and wide-scale socioeconomic disruptions and human impacts (including famines made worse by mismanagement, often claimed to be deliberate^20^) across several eighteenth to twentyteeth century colonial societies^20,41^. Our observations suggest that drier climate cycles, especially in a warming climate, appear to be prone to a rainfall reduction threshold of ~ 1 SD, which colonial economic practices and policies may have exacerbated, leading to extreme negative socioeconomic disruptions that came at a steep human cost^20,21^.

Finally, we find that the famines associated with the ~ 1SD reduction in rainfall, which occur more frequently during the last decades of the nineteenth century (marking the transition of the LIA to the warmer climate regimes of the twentyteenth century), had a return period between 5 and 10 years in the Indian peninsular SARs presidencies (Figs. 3–4, S3-S5). The return periods of the famines could reflect low rainfall episodes caused by a modulation of El Nino Southern Oscillation (ENSO) cycles by the Indian Ocean Dipole or the IOD^28–30^ (Figs. S2-S5); warm phases of ENSO and IOD as well as amplification of ENSO cycles due land–atmosphere feedbacks^28–32,45^ are associated with increased frequency of low rainfall (droughts) in semi-arid regions of the southern parts of peninsular India.

## Conclusion

In conclusion, we find that small rainfall reductions (≥ 1 SD but never exceeding 3 SD from regional long-term annual average rainfall totals in one year) were associated with large-scale and often extreme socioeconomic disruptions starting with crop failure and food grain price hike (famines). When the reduction in rainfall continued for a second year, especially if accompanied by a reduction in both summer and winter rainfall, cascading socioeconomic impacts led to extreme human costs (starvation, death, slavery and migration being most prominent). However, during none of the instances of famine in the SIS during the eighteenth and nineteeth centuries, did regional annual precipitation reduce more than 3 SD i.e. extreme rainfall deficits were not a necessary condition for famines in the region. The term ‘famine’ does not appear in twentieth century archived British administrative documents at the NAI after the establishment of the last report by the Famine commission nor in the policy literature of post-1947 independent India^21^. Furthermore, over the second half of the twentieth century to the present decade of the twenty-first century, although we observe low rainfall episodes in southern Indian SARs have exceeded the 1 SD threshold on several occasions, namely 1905–1906, 1908–1924, 1937–1945 during colonial administration and 1972, 1982–1990, 1997–2004, 2011–2015 in post 1947 independent India (Fig. S6), all of which were associated widescale economic and human impacts^6,7,9–12,47^, but not of ‘famine’ proportions. Finally, our record of socioeconomic impacts associated with rainfall fluctuation in semi-arid southern India during the colonial period indicates that assessments of climate risk should consider the potential impacts of more frequent low-level anomalies (of ~ 1 SD) in SARs that are at heightened risk from climate variability.

## Supplementary Information

Supplementary Information.
