# Supplementary material for: Extreme rainfall deficits were not the cause of recurring colonial era famines of southern Indian semi-arid regions
Source: Sci Rep. 2021 Sep 2;11:17568. doi: 10.1038/s41598-021-96826-2 (PMC8413344; doi:10.1038/s41598-021-96826-2)
Supplement: Supplementary file 1 — Supplementary Information. [file 41598_2021_96826_MOESM1_ESM.pdf]

Supplementary Information (SI)

Section A: Description of indirect (proxy) climate data

1. **18th-19th century tree ring based reconstructions of rainfall**
2. **Long term rainfall climatology based on high-resolution reconstructions from calcium carbonates of cave deposits**

Section B: Description of presidencies

Section C: Digitized document

Section D: Table of results

Section E: Figures

Section A: Description of indirect (proxy) data

1. **18th-19th century tree ring based reconstructions of rainfall climatology:** A 19th-20th century tree ring based reconstruction of rainfall climatology of peninsular India (Fig S4): Regional tree ring research over the past few decades has demonstrated that quantitative reconstruction of past monsoonal climate over the period of few centuries is possible through dendroclimatology (24-25). We have plotted tree ring width data from Edugurapalli in Telengana, Allapalli, Telengana and Bori, Madhya Pradesh (24-25), which lies at the northeastern part of Madras Presidency. Annual tree ring widths are longer during high rainfall years and shorter on low rainfall years

2. **Long-term rainfall climatology based on high-resolution reconstructions from calcium carbonates of cave deposits (speleothems):** Long term high-resolution rainfall climatology is presented in Fig. 4 and S5. Oxygen isotopes in speleothem in cave deposits record climatology w.r.t rainfall; we used oxygen isotope data from stalagmites collected by (26-27) from Jhumar Cave (18° 52′N, 81° 52′E; 600 masl) located near the town of Jagdalpur in central India. The amount effect is the observed decrease in rainfall $\delta^{18}O$ values with increased rainfall amount ($\delta^{18}O_p/dP$) (46,48). The ECHAM-4 AGCM model fitted with isotope tracers to explore the amount effect across the Asian monsoon region and confirm that there is a negative correlation between $\delta^{18}O$ of precipitation and monsoon circulation from the Bay of Bengal across the central Indian subcontinent (49); the authors demonstrate that in central India, stronger sea to land circulation generates increased monsoon rainfall, and subsequently a measureable amount effect. As such, $\delta^{18}O$ changes in Jhumar Cave $CaCO_3$ primarily reflect variations in monsoon season rainfall amounts. Theoretical estimates of cave's ambient temperature, calculated from $\delta^{18}O$ measurements in dripwater and modern calcite from both chambers, are within the range of measured temperatures, suggesting that precipitation of calcite in the cave is occurring in isotopic equilibrium (26-27, 46, 48-49).

Section B

[I] History of the Madras and Bombay Presidencies: Peninsular India, like most parts of the subcontinent, was under British colonial rule between the late 17th century to the mid 1900s; in 1600, the British sovereign, Queen Elizabeth I granted a group of English merchants the permission to form a company known as the East India Company (EIC), which established two presidencies to govern EIC activities in peninsular India, namely the Madras Presidency in 1652 and the Bombay Presidency in 1661 (42). After the Anglo-Mysore Wars (1766–1799), the Anglo–Maratha Wars (1775–1818), both presidencies expanded to their final structures by the early part of the 18th century (43). The Madras Presidency included areas represented by present day states of Tamil Nadu, Andhra Pradesh parts of Orissa, Kerala, Karnataka and the union territory of Lakshadweep. The Bombay Presidency included areas represented by present day states of Maharashtra, Gujarat, parts of Karnataka and parts of Pakistan (43). Both the presidencies were governed by EIC till 1857. In 1858, following an uprising (the Indian Rebellion of 1857) in the ranks of the Indian army maintained by the EIC, both presidencies came under the jurisdiction of the British government under the sovereign, Queen Victoria (43). Government of India Act 1858 provided for liquidation of East India Company, and transferred the powers of government, territories and revenues to the British Crown. As such the colonial period can be divided into two periods Company period (before 1858) and the Crown period (1858-1947); India obtained independence in 1947 (43).

[II] Relevant work using British Colonial records: English East-India Company (EEIC) systematically made weather observations and collected the weather reports; these are preserved in the British Library. Inspection of those archives revealed 900 log-books of EEIC shSPS containing daily instrumental measurements of temperature and pressure, and 15 subjective estimates of wind speed and direction, from voyages across the Atlantic and Indian Oceans between 1789 and 1834. Those records have been extracted and digitised, providing 273000 new weather records offering an unprecedentedly detailed view of the weather and climate of the late eighteenth and early nineteenth centuries (16, 33-36,50-55). In peninsular India George Adamson and his co-workers reconstructed rainfall and studied the social responses of western India from 1781-1860 using EIC records, private diaries and newspapers from India Office Archives, housed in the British Library, St. Pancras, London, UK and from the archives of the Government of Maharashtra, located at Elphinstone College, Mumbai, India (16, 33-35). Similarly, Walsh et al. 1999 (36) reconstructed rainfall using records written in German of the Royal Danish Lutheran–Protestant Mission now kept in the Archive of the Francke, a charitable missionary organization founded in 1695, in Halle Germany.

Section C. Examples of digitized archival documents

The National Archives (NAI) of India is the custodian of the records of the enduring value of the Government of India. Established on 11 March, 1891 in Calcutta (present day Kolkata) by the British Government as the Imperial Record Department; the NAI was transferred to the new capital, New Delhi, in 1911. K. R. Narayanan, then President of India, declared the "Museum of the National Archives" open to the general public on 6 July 1998. The NAI is the biggest archival repository in South Asia (56) with holdings since 1748. The languages of the records include English, Arabic, Hindi, Persian, Sanskrit and Urdu, and their materials include paper, palm leaf, birch bark and parchment (56). The records are arranged in four categories: Public Records, Oriental Records, Cartographic records and Microfilms, Manuscripts and Private Papers (56) thus providing access to British administrative records

that provide critical information regarding several aspects of life and governance during the 18th and 20th centuries in South Asia. Below are some digitized documents.

Example1:

Poor Fund, Madras, Copies of correspondence between the Madras Native Poor Fund Committee and the Government of St. Fort George for the relief of the poor, January-August 1807

Home, Miscellaneous, Vol- 293
Public and Commercial Department
Copies to the papers respecting the Native Poors from 6th January to 10th August 1807

10th July, 1807

Submitting a plan for extending relief to the distressful poor with a recommendation in consequence:

"The obvious view of Government in agreeing by proclamation to purchase such cargos of rice as might be tendered until the of October ensuing at the high rate of Pagodas 110 per Garce was to encourage importations to the extent of meeting the threatened distress to the inhabitants of this coast owing to the failure of Rain during the period of the last monsoon."

Thomas Oaks,

Esquire
Fort of St. George
Madras

Example 2

Home, Miscellaneous, 1839-40, no- 464

Extract from the Minutes of Consultation under date the 29th July, 1840
Read the following Extract from the Board of Revenue
Relative to the settlement of the Land Revenue of the District of Vizagapatnam for Fustly 1248
Here enter 18th June 1840, No-23

"The season of the fustly under revised appears to have been generally unfavorable throughout the District but more especially so from the center to the southern boundary in the country situated within those limits the drought, it is stated was so severe that the crops failed entirely and the dry were very deficient."

Example 3

Unfavorable prospects of the season in the Districts of Madras, North Arcot and South Arcot, Home Department Proceedings 1st Feb, 1868

> From W. Hudleston, Esquire, Secretary to the Government of Fort St. George, to E.C. Bayley, Esquire, Secretary to the Government of India
>
> Dated: 14th January, 1868
>
> "I am directed to forward for information the accompanying correspondence, from which the Government of India will learn that prospects of the season are very unfavourable in the Districts of Madras, North Arcot, South Arcot owing to the serious failure of the north east monsoon and that apprehensions of distress among the poorer classes are entertained."

Example 4 (excerpt)

Report on Madras Famine 1876-1878

July 1882, Proceedings 1-6, Revenue and Agricultural Department, Branch- Famine

"The troubles of the Presidency began as far back as the end of 1875 when the partial failure of both the monsoons resulted in short harvests, though not of such a character as to cause any serious anxiety. In the early part of 1876 the unfavourable reports as to the crops received from Madras attracted the attention of the Government of India, and the Government of Madras called for more detailed information from the districts. Meanwhile, on the representations of the Collectors, relief-works of a limited character were opened in Bellary and Cuddapah. The south-east monsoon of 1876 failed in these and other districts on the Dekkan plateau. Matters grew very threatening, and the numbers on the relief-works increased. Towards the end of October 60,000 people were on relief-works in Bellary alone, and over Rs. 3,00,000 had already been spent on wages. Prices rose with alarming rapidity: coarse rice was selling at 7 and 8 seers, and ragi between 8 and 10. All hope of the south-east monsoon was by this time over, and the north-west monsoon proved an equal failure. In Nellore the rainfall was less than 2 inches against an average of 21, in Chingleput 4 against 24, and in Trichinopoly 3 against 17. In Bellary, Cuddapah and Salem the aspect of the country was as burnt and bare as it usually is in March when the harvest has been reaped; and the cattle died by hundreds for want of fodder.

In the extreme south the failure of the north-west monsoon of 1876 brought Madura and, to a less extent, Tinnevelly into the list of famine districts, and towards the close of 1877 the high price of food and the number on relief-works in the former district indicated great distress. Large stocks of grain were thrown into it from Tanjore and Negapatam by the South Indian Railway, which thus played a very important part in the scheme of relief. Between the months of August 1876 and December 1878 the Madras Railway carried nearly one million tons of grain, and the South Indian Railway nearly 400,000 tons, to the distressed districts."

Section D: Table S1

Year	Area	Climate disasters and their impacts	Reference Number of the source document
1729-33	Madras Presidency	Rain failure causing famine due to crop failure, grain price rise, scarcity	Home, Public-13th Feb 1869, No. 115-127
1786	Tanjore	No mention of rain failure; famine due to crop failure because of farmer migration during Anglo-Mysore War	Microfilms, Madras Presidency –Minutes and Letters Vol. 1 1786-87
1792	Madras Presidency	Rain failure causing famine due to crop failure, grain price rise, scarcity	Home, Public-13th Feb 1869, No. 115-127
1803-07	Madras Presidency (esp. Tanjore, Arcot)	Rain failure causing famine due to crop failure, grain price rise, scarcity	Home, Public-13th Feb 1869, No. 115-127
1803	Bombay Presidency (esp. Khandesh)	Rain failure causing famine due to crop failure, grain price rise, scarcity	Home, Public- 5th Nov 1838, No. 21-23
1803-07	Madras Presidency	Rain failure causing famine due to crop failure, grain price rise, scarcity	Home, Miscellaneous-January-August 1807, No. 293
1811-12	Madras Presidency (esp. Madurai)	Rain failure causing famine due to crop failure, grain price rise, scarcity	Home, Public-13th Feb 1869, No. 115-127
1823-24	Madras Presidency (esp. Masulipatnam, Carnatic and Western districts)	Rain failure causing famine due to crop failure, scarcity, starvation, selling of children and riots.	Home, Public-13th Feb 1869, No. 115-127
1832-33	Madras Presidency	Rain failure causing famine due to crop failure,	Home, Public-13th Feb 1869, No. 115-127

		grain price hike, starvation, migration and death	
1838-40	Madras Presidency (esp. Rajahmundry, Masulipatnam, Guntoor, Nellore)	Rain failure causing famine due to crop failure, grain price rise and scarcity.	Home, Miscellaneous- 1839-40, No. 464
1839	Madras Presidency (esp. Guntoor, Madras)	Rain failure causing famine due to crop failure, grain price rise and scarcity.	Revenue Agriculture, Famine- 11th Nov 1839, No. 8
1839	Bellary, Cuddappah (Madras Presidency)	Rain failure causing famine due to crop failure, grain price rise, scarcity	Home, Public- 27 May 1839, No. 8-10
1845	Bombay Presidency (esp. Khandesh, Surat)	Rain failure causing famine due to crop failure, grain price rise, scarcity of food and water, problem in alternate employment.	Home, Public- 13 Dec 1845, No. 24-6
1854	Petty Hill farms (Madras Presidency)	Forest land transforming into agricultural land	Home, Public- May 1854, No. 3-5
1854-55	Madras Presidency	Rain failure causing famine due to crop failure, grain price rise, scarcity and cattle deaths	Home, Public-13th Feb 1869, No. 115-127
1858	Not mentioned	Excessive denudation.	Home,Medical- 9th Dec 1858, No. 5
1862	Bombay Presidency (esp. Eastern districts and Khandesh)	Rain failure causing famine due to crop failure, grain price rise and scarcity.	Home, Public-7th Nov 1862, No. 2-3 (A)
1862	Ahmednagar, Khandesh (Bombay Presidency)	Rain failure causing famine due to crop failure, grain price rise and scarcity.	Home, Public- 4th October 1862, No. 2-3

1862	Bombay Presidency	Rain failure causing famine due to crop failure, grain price rise and scarcity.	Home, Public- 4th October 1862, No. 4
1862	Ahmednagar (Bombay Presidency)	Rain failure causing famine due to crop failure, grain price rise and scarcity.	Home, Public, A - 17th October 1862, No. 17-19
1868	Not mentioned	Denudation due to rail line construction.	Home, Public- 17th April, 1865, N—60 (A)
1868-69	Madras Presidency (esp. Madras, Arcot)	Rain failure causing famine due to crop failure, well getting dried, no fishing.	Home, Public- 1st Feb 1868
1868-69	Bombay Presidency (esp. Mahabaleshwar, Khandesh)	People are weak, stricken by fever, crowding over the frontier.	Home, Public- 20th Nov 1869, No. 326-327 (A)
1875-78	Madras Presidency and Bombay Presidency	Rain failure causing famine, crop failure, starvation, death, migration, conflict, slavery and diseases.	Home, Public- Dec 1877, No. 222 (B); Home, Public- Jan 1877, No. 90 (B); Revenue and Agricultural Department, Famine- July 1882, No. 1-6; Home, Public- Dec 1889, No. 85-92 (A)
1877	Madras Presidency	Rain failure causing famine, need of medical assistance.	Home, Medical- Oct 1877, No. 67-72 (B); Medical-Aug 1878, No. 69 (B)
1882	Mysore (Madras Presidency)	Rain failure causing crop failure, scarcity of food and drinking water.	Home, Public- April, 1882, No. 5-8
1896	Madras Presidency and Bombay Presidency	Rain failure causing famine due to crop failure, starvation, death, migration, conflict, slavery and diseases.	Home, Public- Sept 1901, No. 90-91 (B); Home, Medical- Nov 1902, No. 57-58 (A)
1899-1900	Madras	Rain failure causing	Home, Public- Sept 1901,

	Presidency and Bombay Presidency	famine due to crop failure, starvation, death, migration, conflict, slavery and diseases	No. 90-91 (B); Home, Medical- Nov 1902, No. 57-58 (A)

Table S1: Record of low rainfall episodes ('rain failure') as well as socioeconomic and human impacts ('famines') of 18th-19th century, extracted from British administrative documents preserved in the National Archives of India, New Delhi, India (56). No institutional documents in the NAI after 1901 report occurrence of famines.The colonial era subsistence crops included Jowar (Millet), Bajra (Pearl Millet) and Wheat, whereas the cash crop was primarily cotton. The water requirement for the crops are as follows: Jowar (250-300 mm, 57), Bajra (150-350 mm, 58), Wheat (450-650 mm, 59) and cottton (500-750 mm, 60).

Section E: Figures

Figure captions

Figure S1: Overall climatology of peninsular India (our study area). Annual precipitation of southern India, which depicts the rain shadow areas comprising the semi-arid regions (SARS) of southern India; the SARS is bounded along the west by the Western Ghats (also known as the Shyadris), which blocks off most of the southwest arm of the monsoon during the rainy season (June-July-August-September) and by the Balaghat ranges in the north, that catch the leftover moisture causing some rainfall in the northern part of the SARs. Because of these features, there is a clear rainfall gradient from the coasts to the interiors. As Chennai (previously Madras), the capital of the state of Tamil Nadu lies along the eastern coast, land and sea breeze systems tend to modify the monsoonal systems; as such, Chennai receives more rainfall compared to the interiors of Tamil Nadu (even though the city lies in the semi-arid regions of southern India).

Figure S2: 21st and 20th century climatological analysis of the study area (based on rainfall data from the Indian Meteorological Division i.e. the IMD). [A] Sidebar shows the Indian Meteorological (IMD, 22) subdivisions of India. In our study, we define two regions, namely interior western India and interior southern India. The 'interior western India' includes Madhya Maharashtra (IMD division 24), Marathwada (IMD division 25) and North Interior Karnataka (IMD division 33) and the 'Interior Southern India' includes Rayalaseema (IMD division 30), Tamil Nadu (IMD division 31) and south interior Karnataka (IMD division 34) and the 'Coastal Western India' includes Konkan and Goa (IMD division 23). The boundaries of interior western India, interior southern India and coastal western India are overlain on the 115-year (1901-2015) precipitation climatology map (IMD,22) of India. As is clear, coastal western India receives substantially higher rainfall compared to the two other divisions of the interior semi-arid regions (SARs) of peninsular India. [B] The 20th century annual average rainfall of interior western India (Madhya Maharashtra, Marathwada and North interior Karnataka), interior southern India (Tamil Nadu, Rayalaseema and South Interior Karnataka) and Coastal Western India (Konkan and Goa) are plotted. Coastal western India (Konkan and Goa) along the west coast i.e. along the sea-facing side of the Western Ghats, receives heavier annual rainfall (almost entirely during June-July-August-September i.e. monsoon season or the JJAS). The western coastal and interior regions receive most of its rainfall

from the southwest monsoons during June-July-August-September (i.e. JJAS); however, southern India receives rainfall both during the monsoon (JJAS) as well as during retreating monsoons during the period October-November-December (i.e. OND). Even then, the annual rainfall of interior south India is comparable to the annual rainfall of interior west India, both lying in the rain shadow region of Western Ghats and the Balaghat Ranges. [C-E]: Spectral analysis (multi-taper method or MTM) of annual rainfall data (IMD) of interior western India (Middle Maharashtra, Marathwada and North Interior Karnataka), interior southern India (Rayalaseema, Tamilnadu and South Interior Karnataka) and coastal western India (Konkan and Goa). The highest powers (for n = 115 i.e. 1901-2015 at 95% CI) are 0.135 (7.4 years), 0.305 (3.3 years) and 0.067 (14 years) and 0.42 (2.38 years) for interior western, interior southern and coastal western regions, respectively. The 2-3 year cycles are related to El-Nino southern oscillations (28-30, 45) and the longer (7-14 year) cycles of the interiors possibly reflect modification of oceanic signals by land-atmosphere feedbacks (31-32).

Figure S3: 19th century climatological analysis of the study area based on historical instrumentation rainfall dataset. [A] Sontakke 2008 (23), collated historical instrumental data for the period 1813-2005 for seven zones in India, including the southern (SPI) and western India (WPI), using 316 well-spread station data for pre-monsoon (March-April-May i.e. MAM), monsoon (June-July-August-September i.e. JJAS) and post monsoon (October-November-December i.e. OND) and annual averages.The image is from the following publication. Authors: N.A Sontakke, Nityanand Singh and H.N. Singh; Journal Title: The Holocene; Journal Volume: 18; Issue Number: 7 pp; 1055-1066; copyright @2008 by Sage Journals; reprinted by permission of Sage Publications Ltd. [B] Here we plot the relationship between famine incidences in peninsular India against the historical instrumentation dataset between 1813-1900 (87 years). Famines plotted on the raw annual average rainfall data of the 19th century (18) of peninsular India (WPI and SPI). [C-D]: Spectral analysis of annual average rainfall data of the 19th century (23) of peninsular India (WPI and SPI, respectively). The highest powers (n=87 at 95CI) are 0.21 (4.8 years) for SPI and (n= 67 at 95 CI) 0.4 (2.5 years) for WPI. The 2-5 year cycles in both SPI and WPI are consistent with majority of rainfall in western India accounted for by the coastal western Indian rainfall (Fig S2) and therefore demonstrating the El-Nino Southern Oscillation (ENSO) signal; the SPI on the other hand has significant portions in semi-arid regions of the interiors (Fig S2) and therefore demonstrate the longer cycles-although because Madras (now Chennai) experiences coastal influence and therefore balances some of the influences of the longer climate cycles of the interiors.

Figure S4: 19th century climatological analysis of our study area based on tree ring rainfall reconstructions. [A] Famines plotted on the raw tree ring thickness (24-25) from three locations in the interior, semi-arid regions of peninsular India. [B-C] Spectral analyses of tree ring thickness (proxy for annual average rainfall variations in SARs of peninsular India) from sites at Edugudapallu (representing years 1827-2008 i.e. n=173), Alapalli (representing years 1997-1866 i.e. n=82), and Bori (representing years 1857-2006 i.e. n=90). The highest powers are 0.25 (4 years) and 0.57 (2.12 years), 0.22 (4.5 years) at 95 CI, 0.28 (~4 years) and 0.35 (~ 3 years) at 99 CI for Alapalli and 0.11 (9 years) and 0.37 (~ 3 years) at 95 CI for Bori. The 2-5 year cycle is consistent with ENSO signals, also seen in modern and historical instruments.

Figure S5: 18th-19th century climatological analysis of our study area based on rainfall reconstructions of cave carbonates using oxygen isotopes. Spectral analysis of oxygen isotopes of Jhumar cave speleothem (26-27), located in the state of Chattisgarh (see inset of Fig 4 of the manuscript for location). Note that, unlike the tree ring sites that lie in our study area, the cave deposits lie along the northern border of our study area; high-resolution reconstructions of long-term rainfall climatology from the cave deposits provide the much-needed rainfall climatology beyond that is covered by the tree ring reconstructions. [A] The highest powers are in the range of 0.44-0.48 (~3 years) at 99 CI and 0.41 (~ 3 years), 0.37-0.33 (~ 4 years) at 95 CI. Note that each time step reflects 1.42 units for the entire series. The 3-4 year cycles are reflective of the ENSO signal in the region between 1075-2008 calendar years. [B] If we only use our study period (i.e. 18th and 19th century) then we see an additional power at 0.013 (~55 year) in addition to 0.45 (~2 year). Note that for the 200 years (1710-1910) of our interest, each time step is 0.72 and has been folded into the conversions of the frequencies to time). The 2-4 year cycle once again demonstrates the ENSO influence in the region. The 55-60 year cycle repeats 4 times in our study window (main manuscript Fig 4) and suggests the presence of a long wet and dry multidecadal cycle. [C] Plot of the famine record of the SPS against a 330-year speleothem record (61) from the IMD Western Coastal Region section. Although the location of the speleothem is not in the SPS, we still find that the famines occurred with rainfall lows that are regionally coherent.

Figure S6: 20th and 21st century drought record of peninsular India. The IMD (22) rainfall of (Fig S2) of the three sub-divisions, namely interior western India and interior southern India is plotted. As mentioned in the manuscript the term famine did not appear in the British Administrative documents of the first half the 20th century and did not reappear in the Indian administrative documents thereafter. We also observe that the 14% (1 SD) threshold has been breached on several occasions drought related impacts were mentioned (e.g. 1908-24, 1937-45, 1982-90, 1997-2004 and 2011-15, 8-13,46).

Additional References

48. Dansgaard, W. Stable isotopes in precipitation. Tellus A: Dynamic Meteorology and Oceanography, 16 (4), 436-468 (1964).

49. Lachiniet, M.S. Climatic and environmental controls on speleothem oxygen-isotope values. Quaternary Science Reviews, 28, 412–432 (2009).

50. Brohan, P., Allan, R., Freeman, E., Wheeler, D., Wilkinson, C., & Williamson, F.: Constraining the temperature history of the past millennium using early instrumental observations, Climate of the Past, 8, 1551–1563 (2012).

51. BráZdil, R., Pfister, C. & Wanner, H. Historical Climatology In Europe – The State Of The Art. Climatic Change 70, 363–430 (2005).

52. Kiss, A. The great (1506–1507) drought and its consequences in Hungary in a (Central) European context. Regional Environmental Change, 20, 50 (2020).

53. Nash, D. J., Endfield, G. H. ‘Splendid rains have fallen’: links between El Nino and rainfall variability in the Kalahari, 1840-1900. Climatic. Change, 86, 257–290 (2008).

54. Nicholson, S. E., Klotter, D. & Dezfuli, A. K. Spatial reconstruction of semi-quantitative precipitation fields over Africa during the nineteenth century from documentary evidence and gauge data. Quarternary Research, 78(1), 13–23 (2012).

55. Damodaran, V. Famine in Bengal: a comparison of the 1770 famine in Bengal and the 1897 famine in Chotanagpur. Medieval History Journal, 10 (1-2), 143-181 (2007).

56. National Archives of India Library, National Archives of India, Janpath Road, New Delhi, India. http://nationalarchives.nic.in

57. Agritech portal, The Tamil Nadu Agricultural University (TNAU), sponsored by the Rashtriya Krishi Vikas Yojana (RKVY). https://agritech.tnau.ac.in/about_us/abt_us.html

58. Ullah, A. and Ahmad, A. and Khaliq, T. and Akhtar, J. Recognizing production options for pearl millet in Pakistan under changing climate scenarios. Journal of Integrative Agriculture, 15, 1-12 (2016).

59. Agaropedia, Indian Institute of Technology (IIT), Kanpur. http://agropedia.iitk.ac.in/

60. Freeland, Jr, T.B., Pettigrew, W.T., Thaxton, P., Andrews, G.L. Agrometeorology and cotton production. World Meteorological Organization. 10(1).1-128 (2011).

61. Yadava, M.G, Ramesh, R., Pant, G. B. Past monsoon rainfall variations in peninsular India recorded in a 331-year-old speleothem. The Holocene, 14(4), 517-524 (2004).

Figure S-1

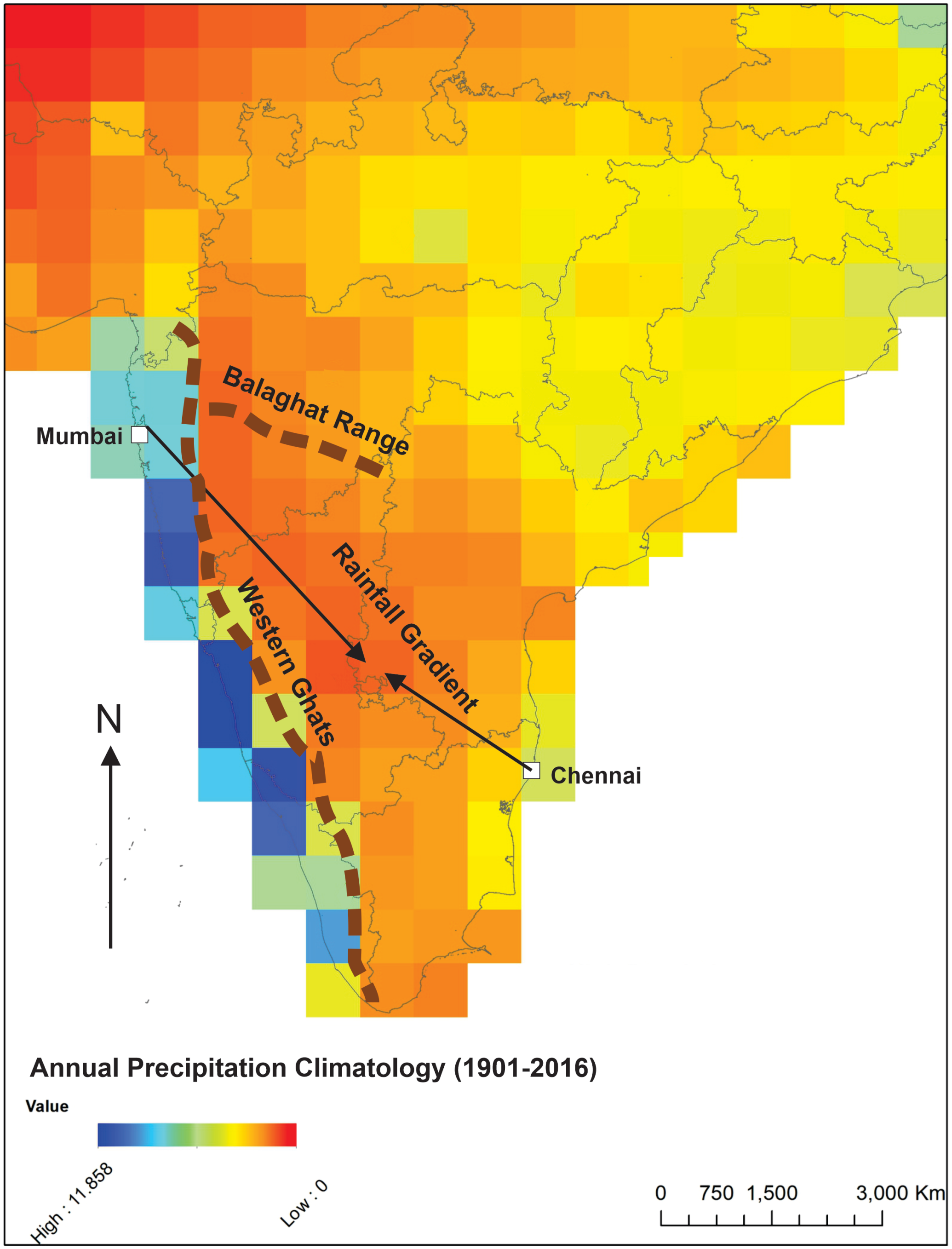
Balaghat Range
Mumbai
Rainfall Gradient
Western Ghats
Chennai
N
Annual Precipitation Climatology (1901-2016)
Value
High : 11.858
Low : 0
0 750 1,500 3,000 Km

Figure S-2A

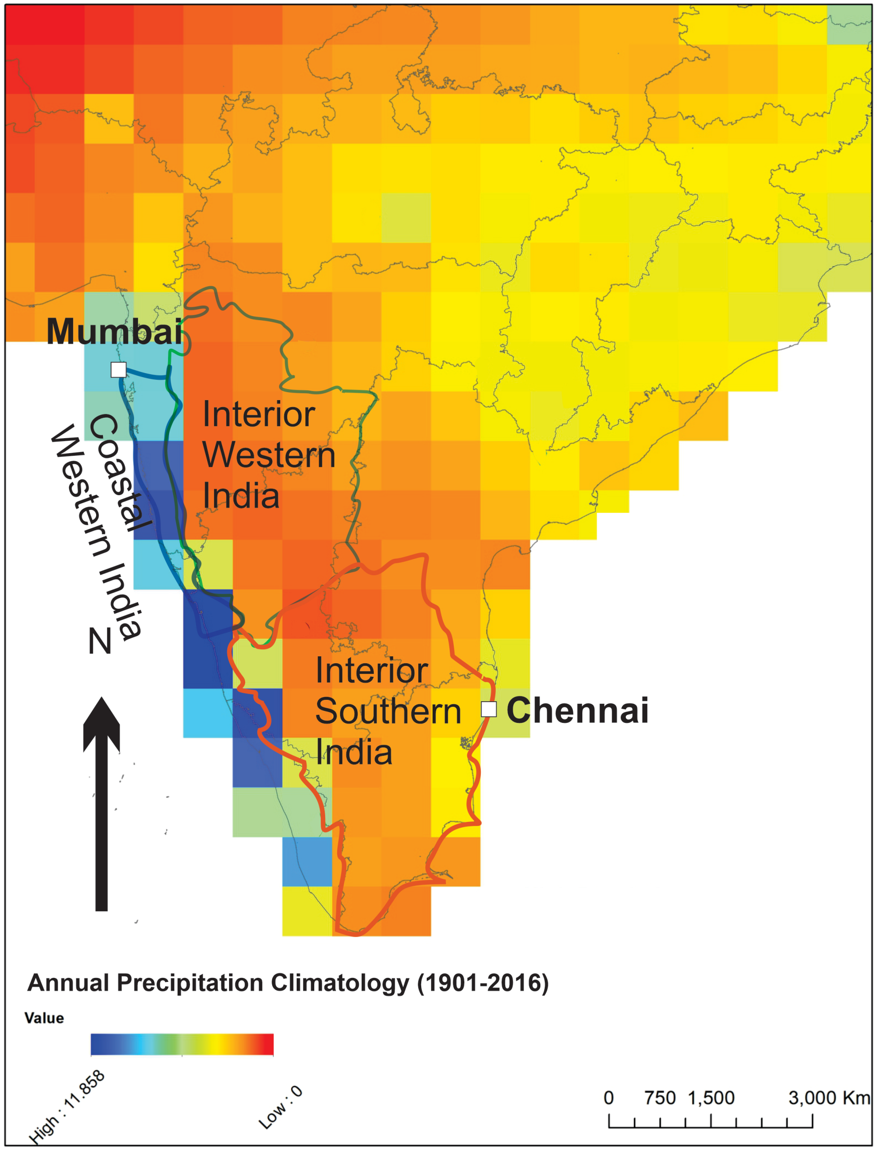

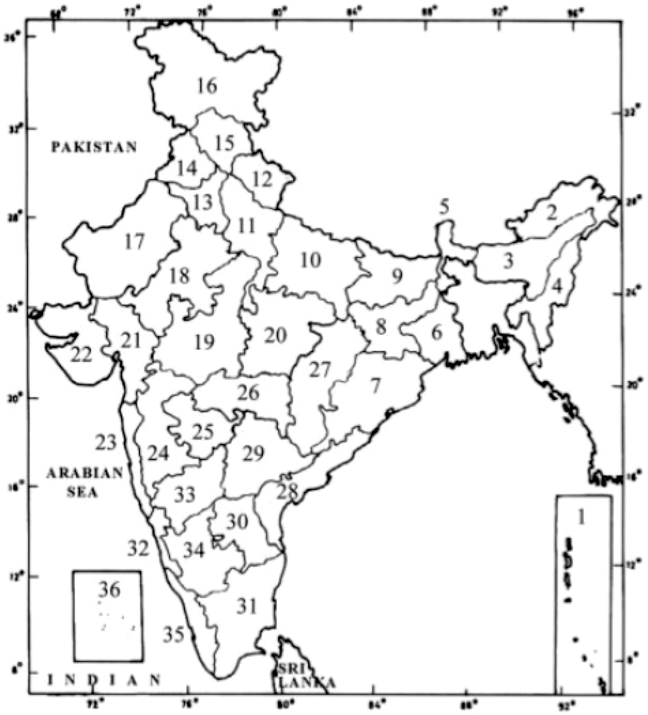

Meteorological Subdivisions of India, IMD

Interior Western India:
24- Madhya Maharashtra
25- Marathwada
33- North Interior Karnataka

Interior Southern India
30- Rayalaseema
31- Tamilnadu
34- South Interior Karnataka

Coastal Western India
23- Konkan and Goa

Figure S-2B

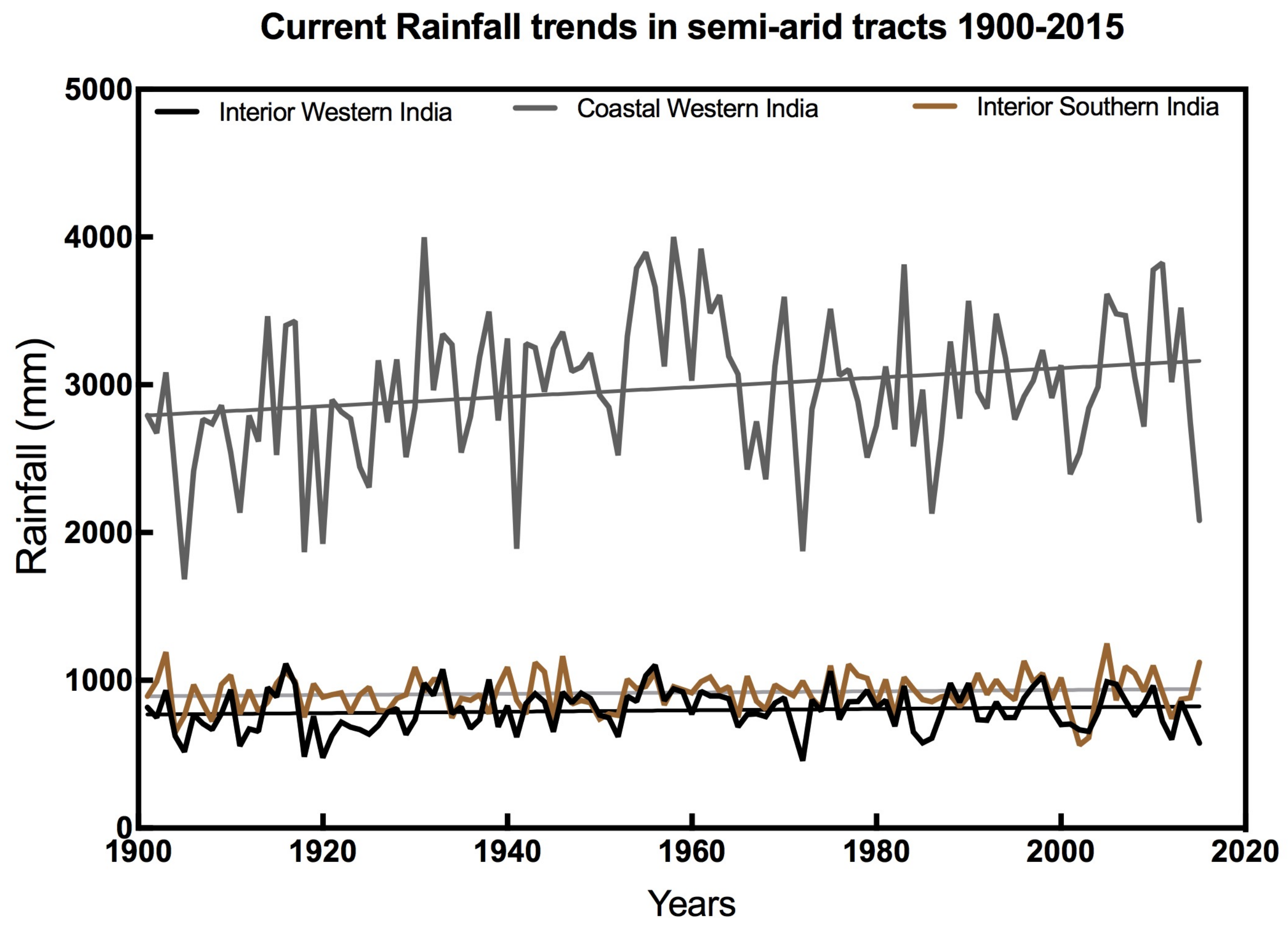

Current Rainfall trends in semi-arid tracts 1900-2015
Interior Western India
Coastal Western India
Interior Southern India
5000
4000
3000
2000
1000
0
Rainfall (mm)
1900
1920
1940
1960
1980
2000
2020
Years

Figure S-2C

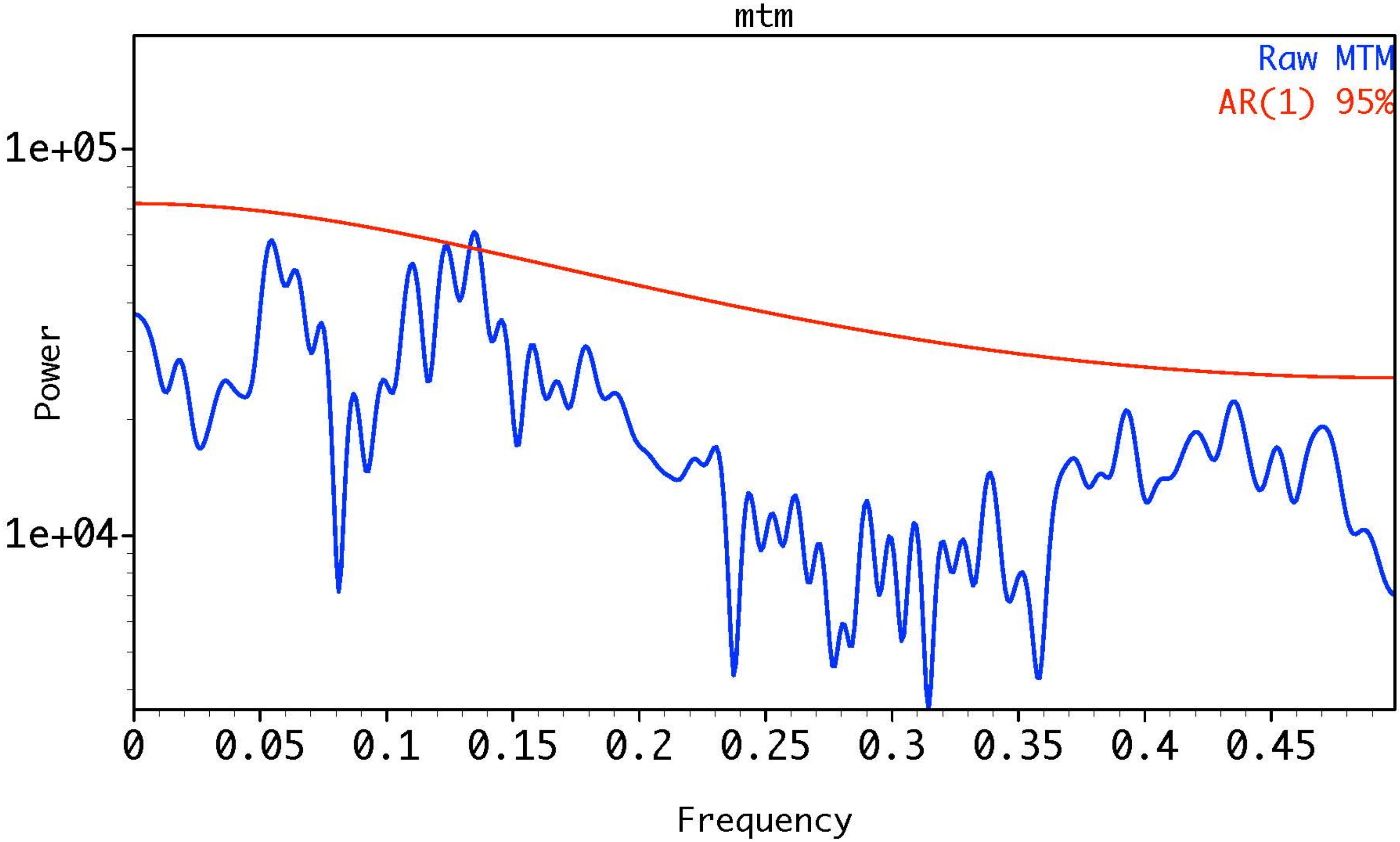

mtm
Raw MTM
AR(1) 95%
1e+05
1e+04
Power
0
0.05
0.1
0.15
0.2
0.25
0.3
0.35
0.4
0.45
Frequency

Figure S-2D

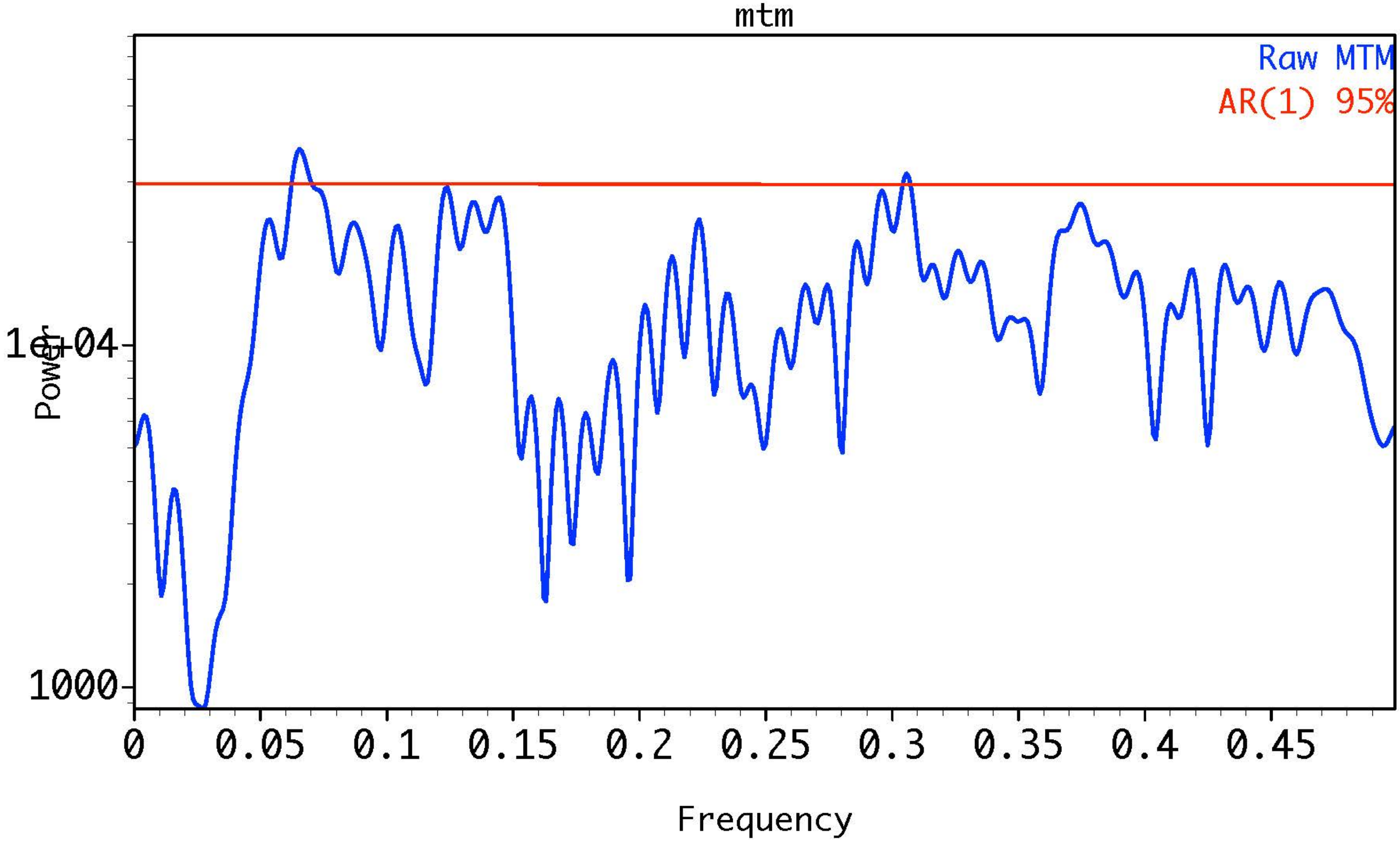

mtm
Raw MTM
AR(1) 95%
Power
1e+04
1000
0
0.05
0.1
0.15
0.2
0.25
0.3
0.35
0.4
0.45
Frequency

Figure S-2E

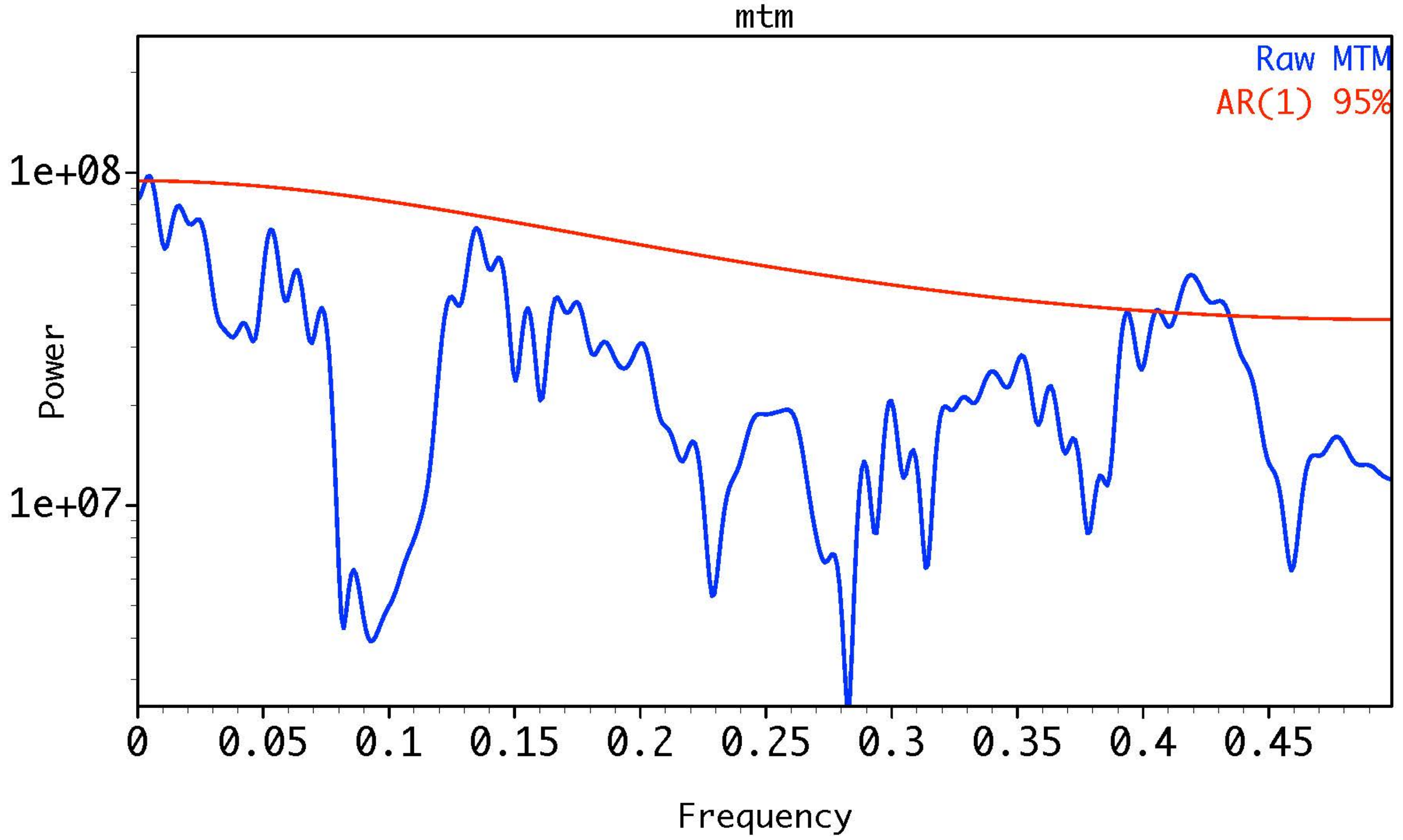
mtm
Raw MTM
AR(1) 95%
1e+08
1e+07
Power
0
0.05
0.1
0.15
0.2
0.25
0.3
0.35
0.4
0.45
Frequency

Figure S-3A

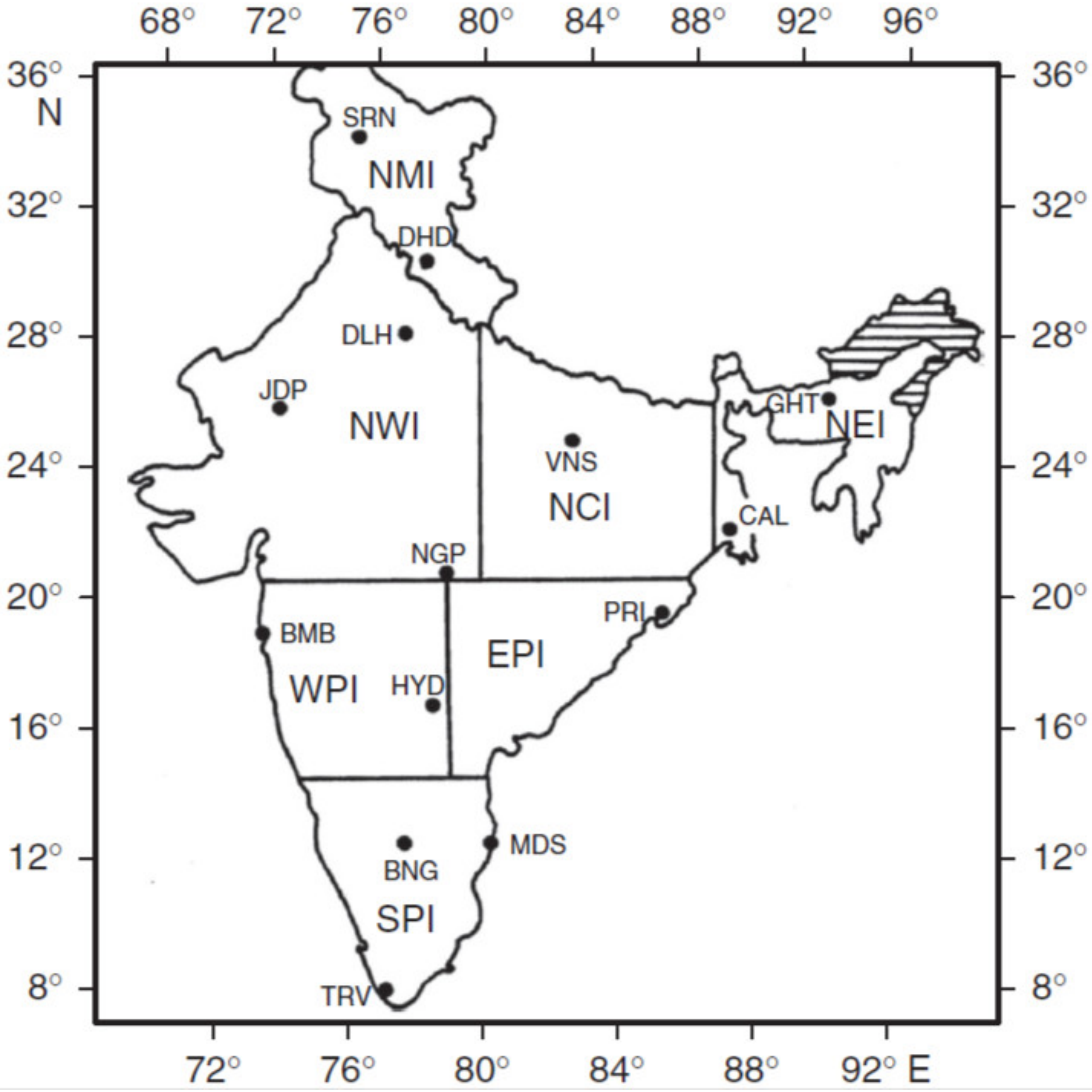

68° 72° 76° 80° 84° 88° 92° 96°
36° N
32°
28°
24°
20°
16°
12°
8°
SRN
NMI
DHD
DLH
JDP
NWI
VNS
NCI
GHT
NEI
CAL
NGP
PRI
BMB
EPI
WPI
HYD
MDS
BNG
SPI
TRV
72° 76° 80° 84° 88° 92° E

Figure S-3B

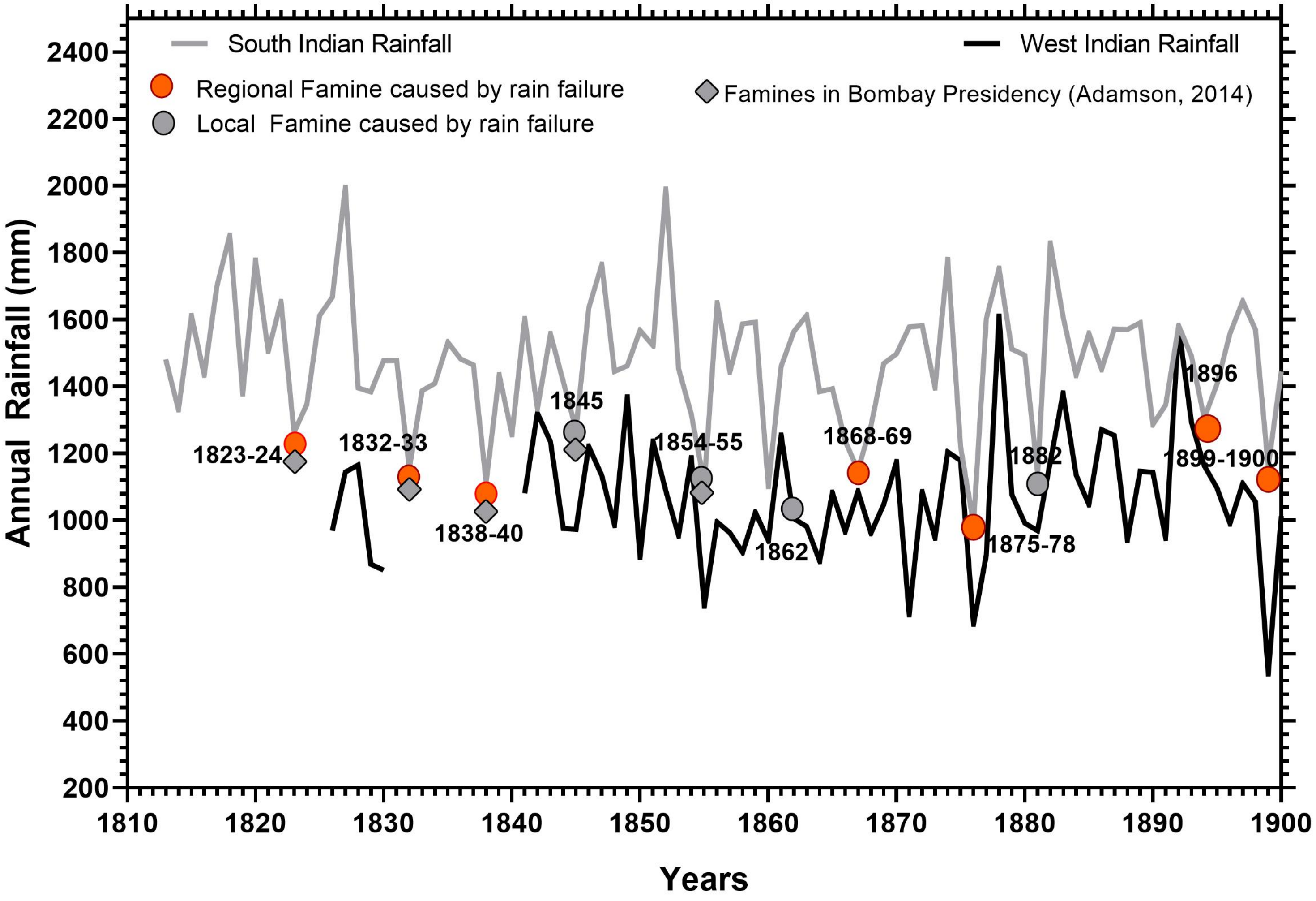

South Indian Rainfall
West Indian Rainfall
Regional Famine caused by rain failure
Local Famine caused by rain failure
Famines in Bombay Presidency (Adamson, 2014)
Annual Rainfall (mm)
Years
1823-24
1832-33
1838-40
1845
1854-55
1862
1868-69
1875-78
1882
1896
1899-1900
2400
2200
2000
1800
1600
1400
1200
1000
800
600
400
200
1810
1820
1830
1840
1850
1860
1870
1880
1890
1900

Figure S-3C

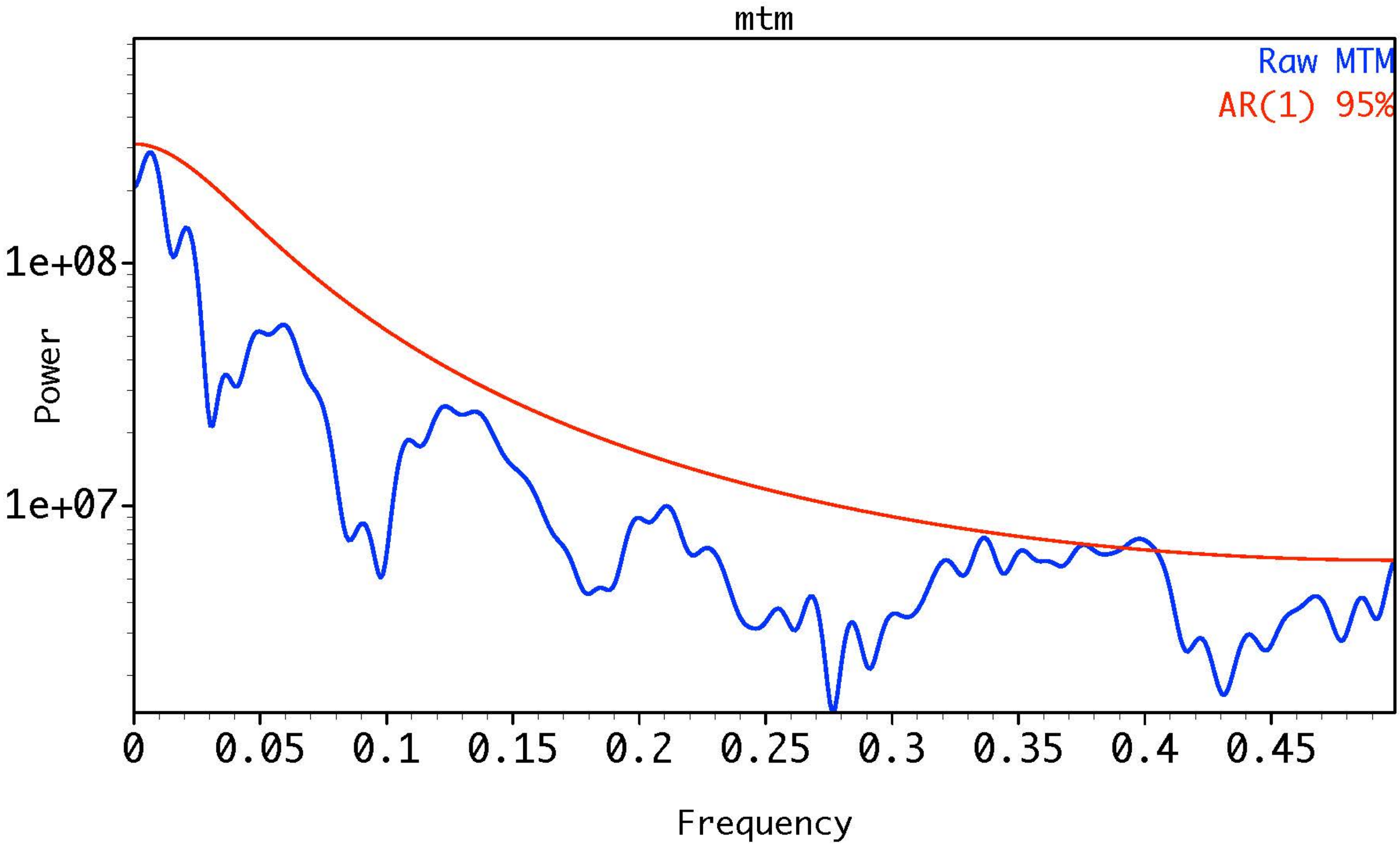
mtm
Raw MTM
AR(1) 95%
1e+08
1e+07
Power
0
0.05
0.1
0.15
0.2
0.25
0.3
0.35
0.4
0.45
Frequency

Figure S-3D

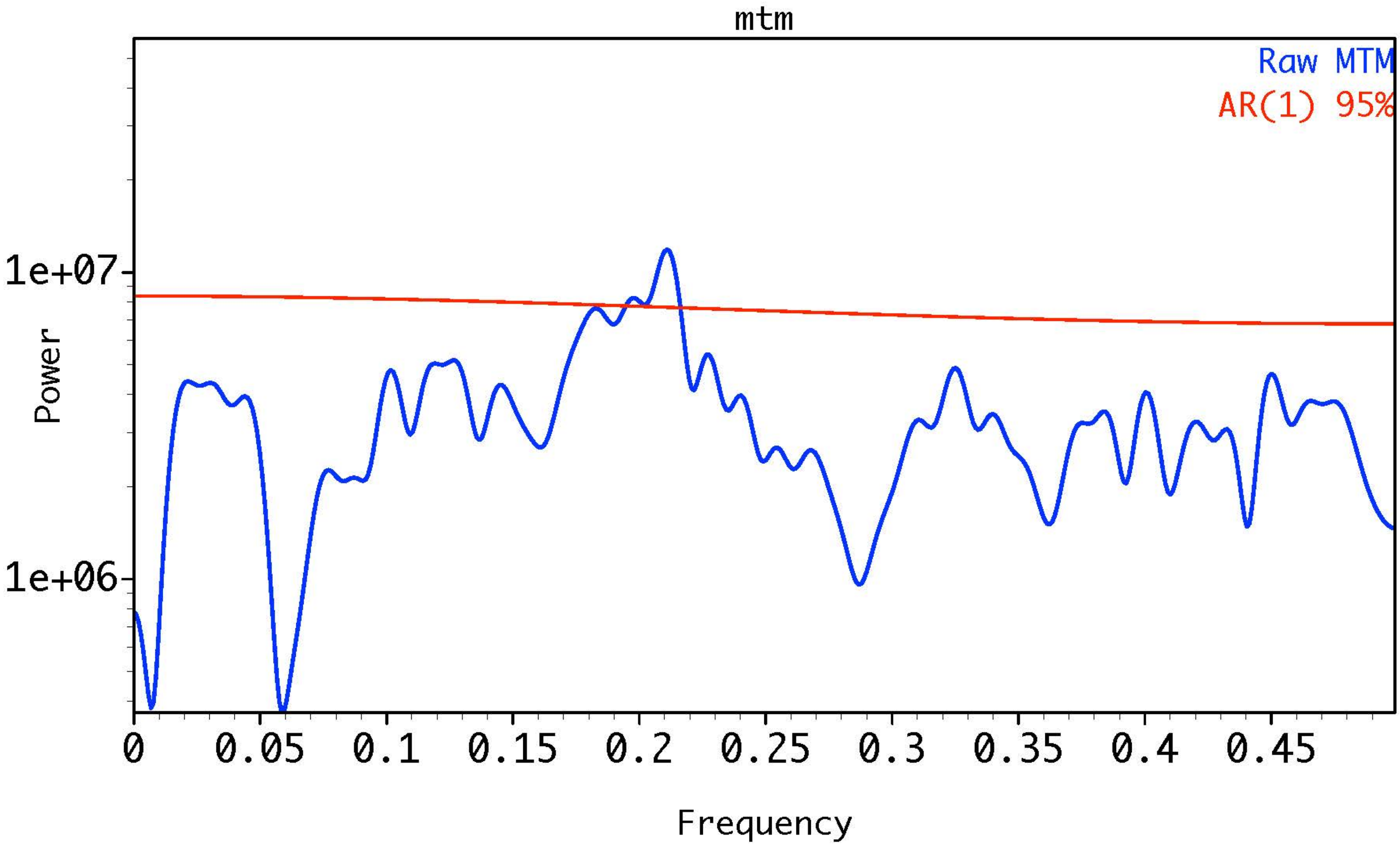
mtm
Raw MTM
AR(1) 95%
1e+07
1e+06
Power
0
0.05
0.1
0.15
0.2
0.25
0.3
0.35
0.4
0.45
Frequency

Figure S-4A

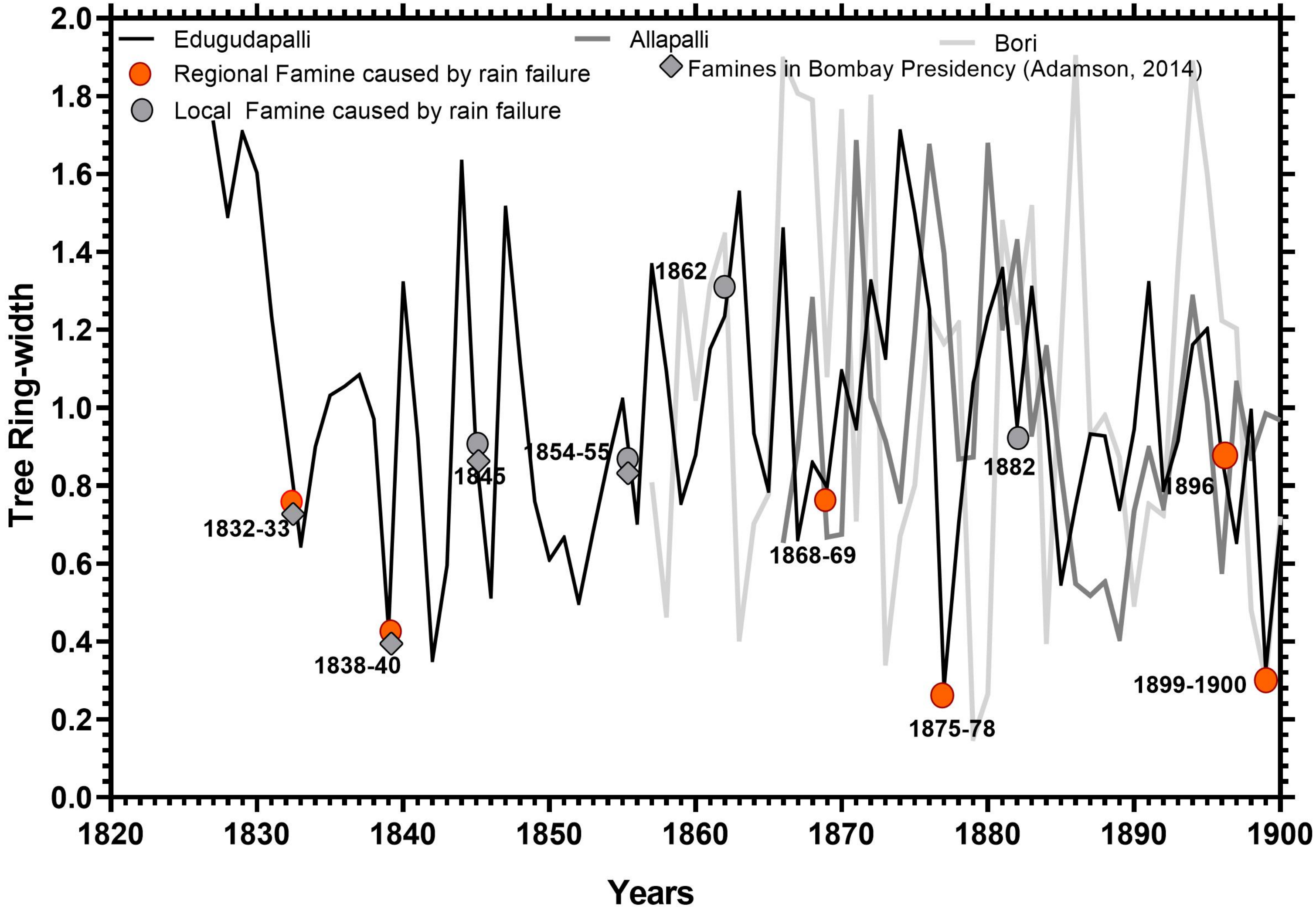
Edugudapalli
Allapalli
Bori
Regional Famine caused by rain failure
Famines in Bombay Presidency (Adamson, 2014)
Local Famine caused by rain failure
Tree Ring-width
Years
2.0
1.8
1.6
1.4
1.2
1.0
0.8
0.6
0.4
0.2
0.0
1820
1830
1840
1850
1860
1870
1880
1890
1900
1832-33
1838-40
1845
1854-55
1862
1868-69
1875-78
1882
1896
1899-1900

Figure S-4B

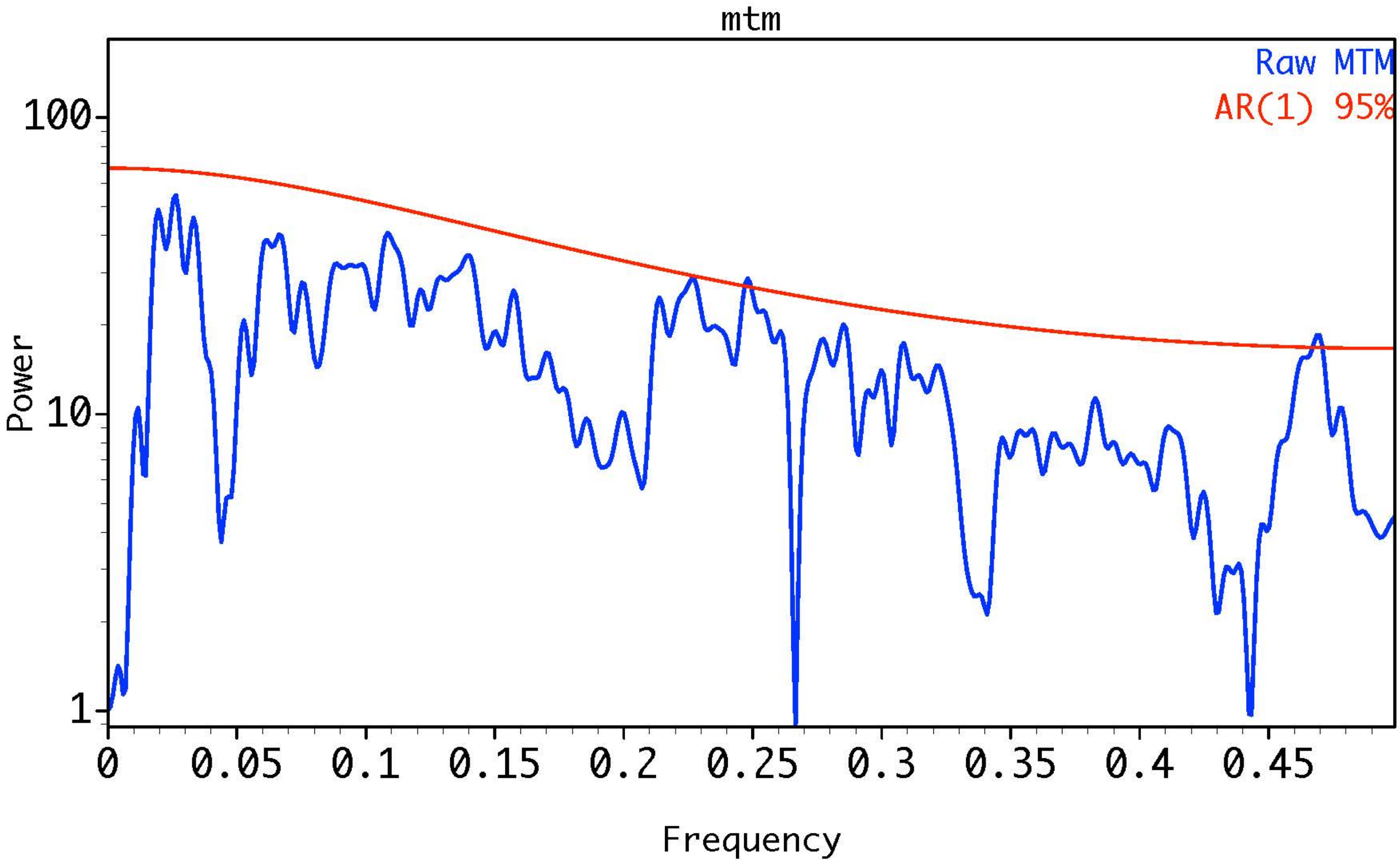
mtm
Raw MTM
AR(1) 95%
100
10
1
Power
0
0.05
0.1
0.15
0.2
0.25
0.3
0.35
0.4
0.45
Frequency

Figure S-4C

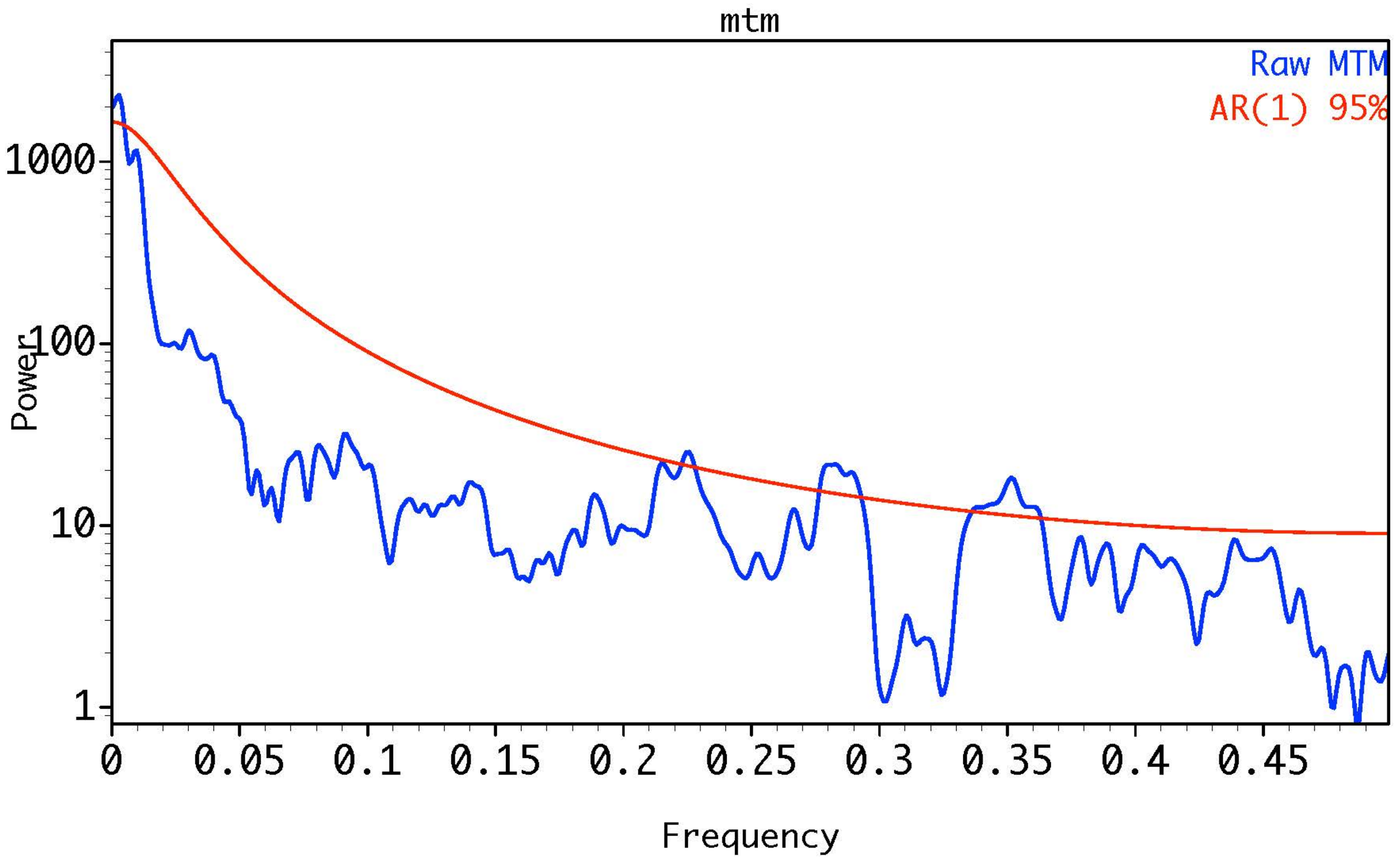
mtm
Raw MTM
AR(1) 95%
1000
100
10
1
Power
0
0.05
0.1
0.15
0.2
0.25
0.3
0.35
0.4
0.45
Frequency

Figure S-4D

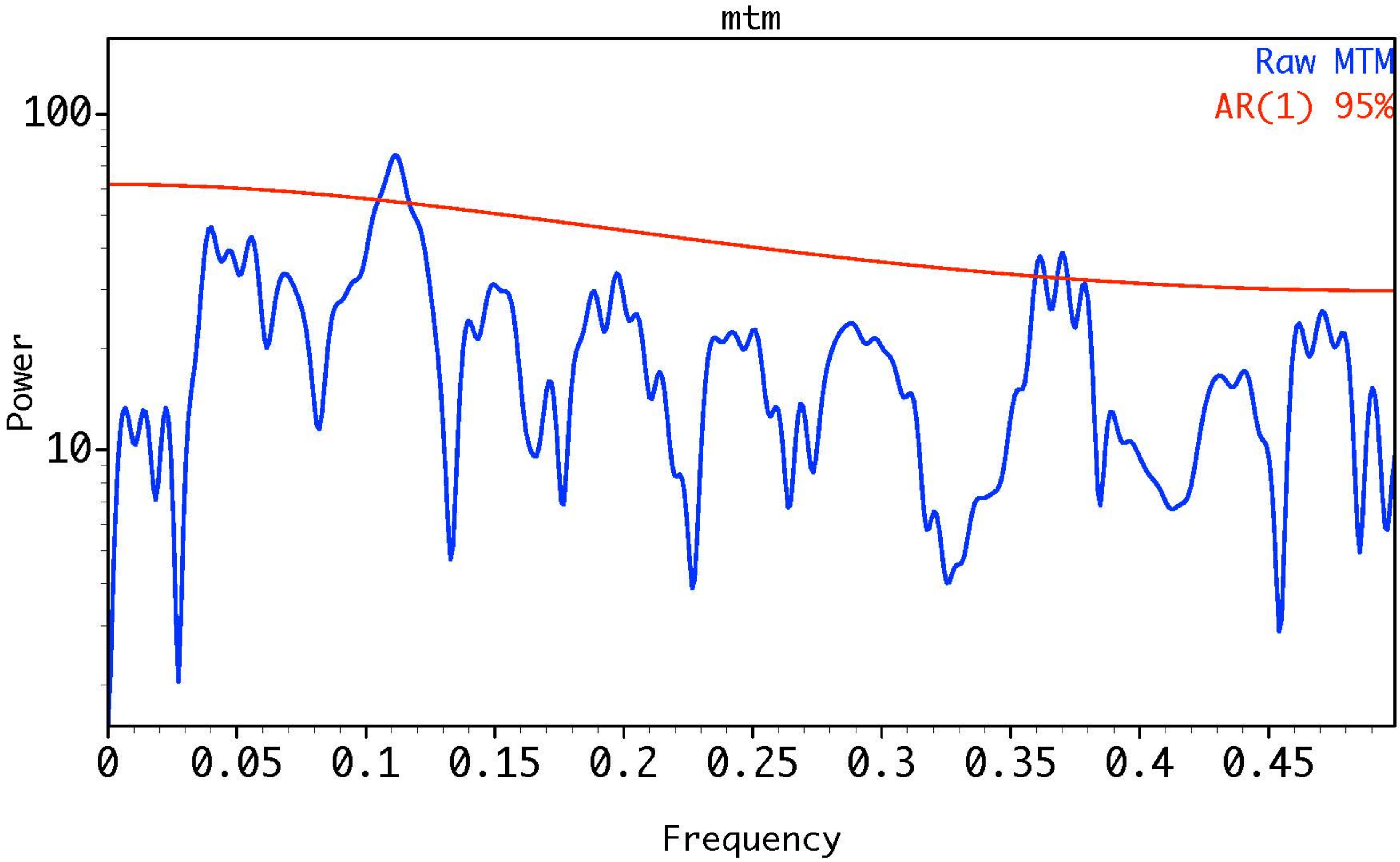

mtm
Raw MTM
AR(1) 95%
100
10
Power
0
0.05
0.1
0.15
0.2
0.25
0.3
0.35
0.4
0.45
Frequency

Figure S-5A

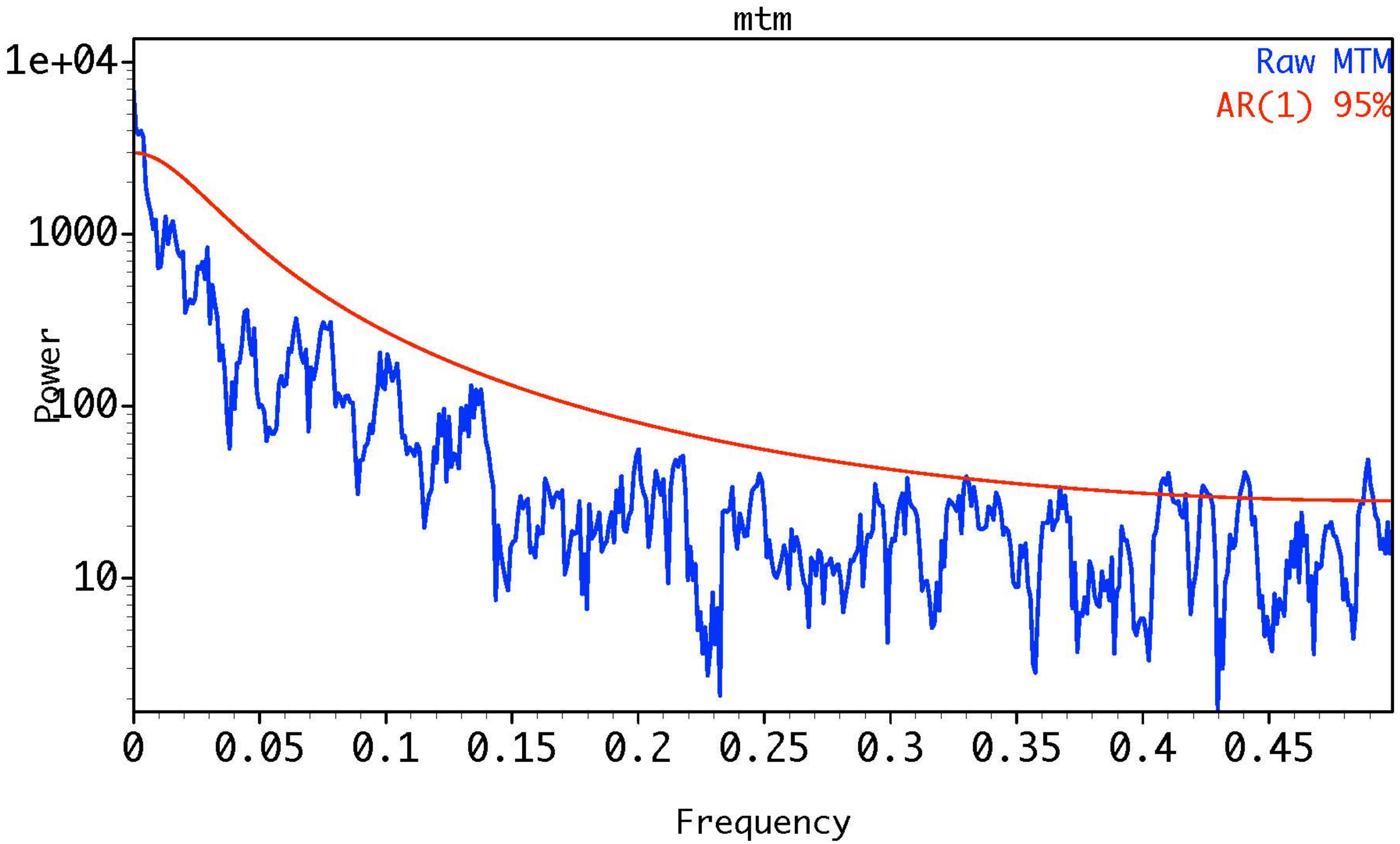

mtm
Raw MTM
AR(1) 95%
1e+04
1000
100
10
Power
0
0.05
0.1
0.15
0.2
0.25
0.3
0.35
0.4
0.45
Frequency

Figure S-5B

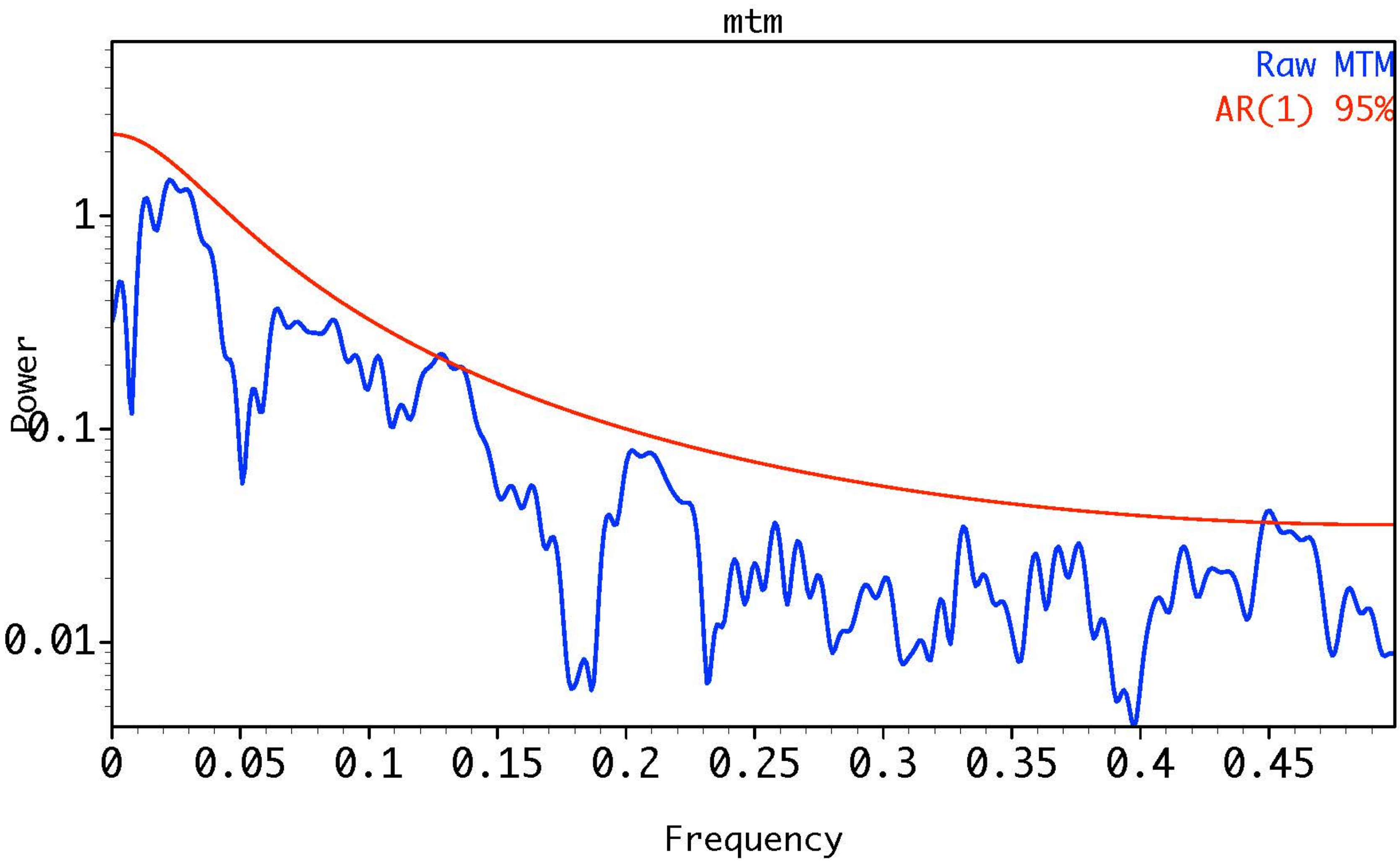
mtm
Raw MTM
AR(1) 95%
Power
1
0.1
0.01
0
0.05
0.1
0.15
0.2
0.25
0.3
0.35
0.4
0.45
Frequency

Figure S-5C

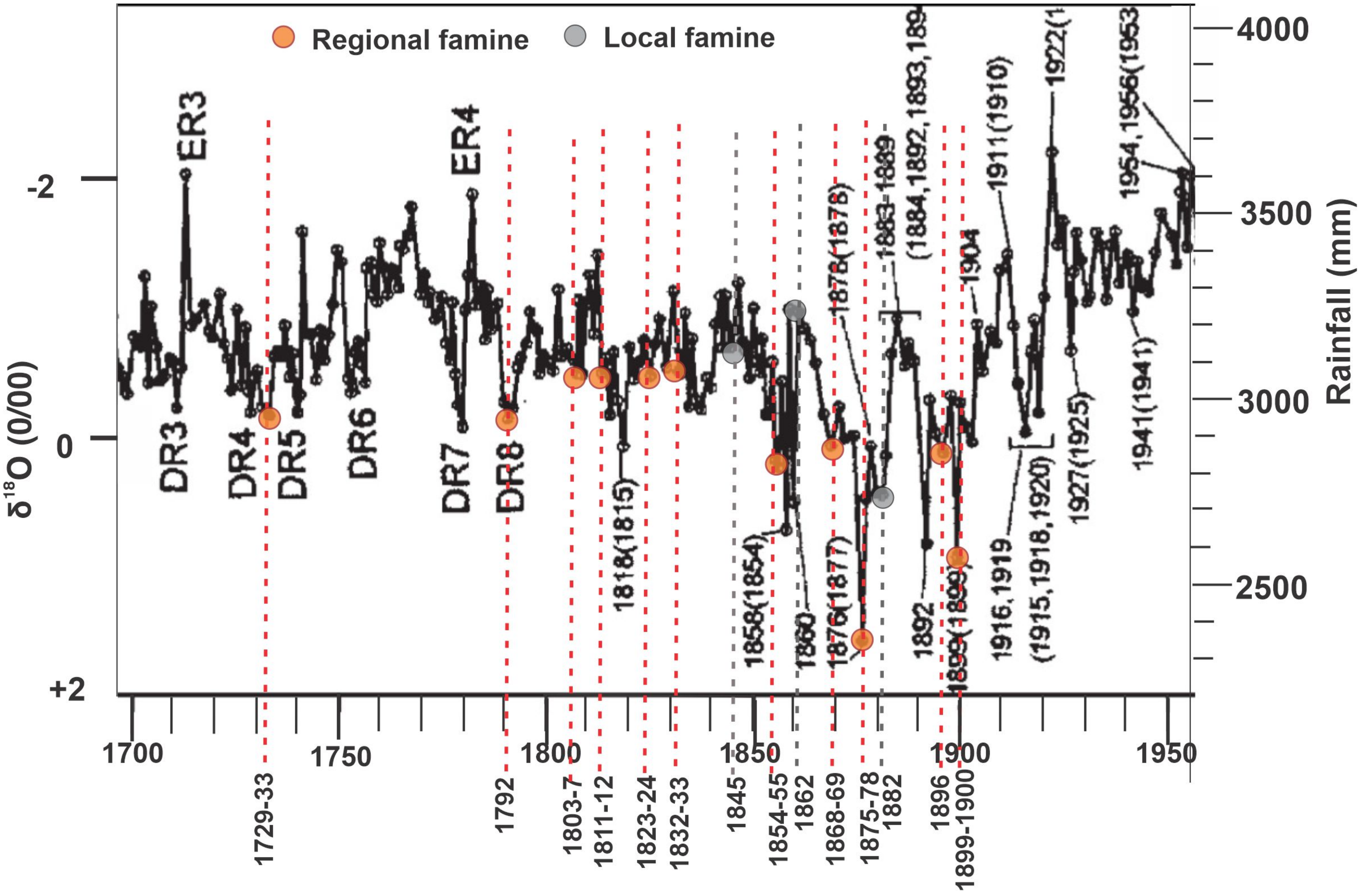

Regional famine
Local famine
δ18O (0/00)
-2
0
+2
Rainfall (mm)
4000
3500
3000
2500
ER3
ER4
DR3
DR4
DR5
DR6
DR7
DR8
1700
1750
1800
1850
1900
1950
1729-33
1792
1803-7
1811-12
1823-24
1832-33
1845
1854-55
1862
1868-69
1875-78
1882
1896
1899-1900
1818(1815)
1858(1854)
1860
1876(1877)
1878(1878)
1883-1889
(1884,1892,1893,189
1892
1904
1911(1910)
1916,1919
(1915,1918,1920)
1922(1
1927(1925)
1941(1941)
1954,1956(1953

Figure S-6

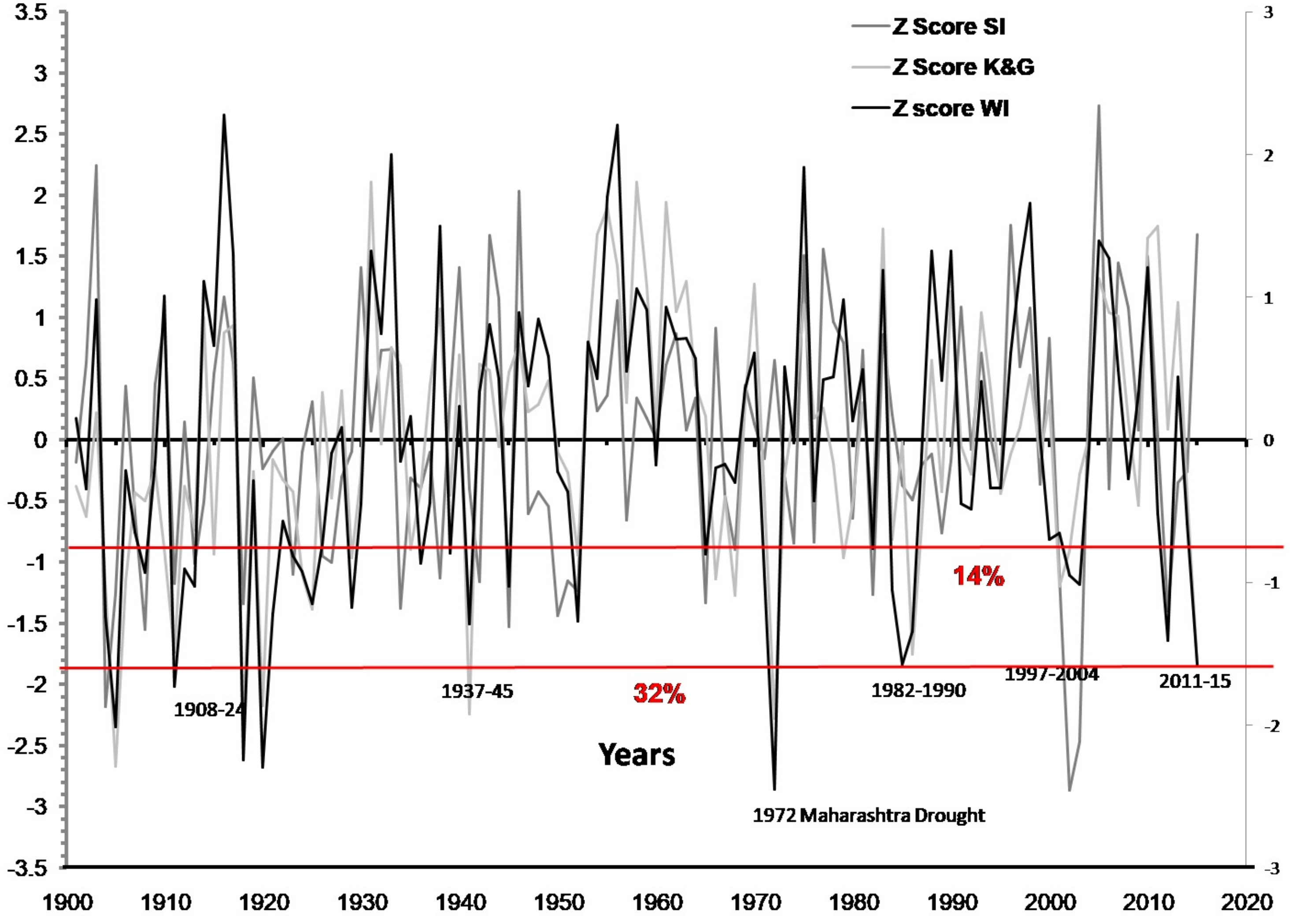
Z Score SI
Z Score K&G
Z score WI
1908-24
1937-45
32%
14%
1982-1990
1997-2004
2011-15
1972 Maharashtra Drought
Years
3.5
3
2.5
2
1.5
1
0.5
0
-0.5
-1
-1.5
-2
-2.5
-3
-3.5
1900
1910
1920
1930
1940
1950
1960
1970
1980
1990
2000
2010
2020
